# Transcriptional Repression and Protein Degradation of the Ca^2+^-Activated K^+^ Channel K_Ca_1.1 by Androgen Receptor Inhibition in Human Breast Cancer Cells

**DOI:** 10.3389/fphys.2018.00312

**Published:** 2018-04-16

**Authors:** Anowara Khatun, Motoki Shimozawa, Hiroaki Kito, Mayu Kawaguchi, Mayu Fujimoto, Moe Ri, Junko Kajikuri, Satomi Niwa, Masanori Fujii, Susumu Ohya

**Affiliations:** ^1^Division of Pathological Sciences, Department of Pharmacology, Kyoto Pharmaceutical University, Kyoto, Japan; ^2^Department of Pharmacology, Graduate School of Medical Sciences, Nagoya City University, Nagoya, Japan

**Keywords:** Ca^2+^-activated K^+^ channel, K_Ca_1.1, antiandrogen, androgen receptor, breast cancer, ubiquitin E3 ligase

## Abstract

The large-conductance Ca^2+^-activated K^+^ channel K_Ca_1.1 plays an important role in the promotion of breast cancer cell proliferation and metastasis. The androgen receptor (AR) is proposed as a therapeutic target for AR-positive advanced triple-negative breast cancer. We herein investigated the effects of a treatment with antiandrogens on the functional activity, activation kinetics, transcriptional expression, and protein degradation of K_Ca_1.1 in human breast cancer MDA-MB-453 cells using real-time PCR, Western blotting, voltage-sensitive dye imaging, and whole-cell patch clamp recording. A treatment with the antiandrogen bicalutamide or enzalutamide for 48 h significantly suppressed (1) depolarization responses induced by paxilline (PAX), a specific K_Ca_1.1 blocker and (2) PAX-sensitive outward currents induced by the depolarizing voltage step. The expression levels of K_Ca_1.1 transcripts and proteins were significantly decreased in MDA-MB-453 cells, and the protein degradation of K_Ca_1.1 mainly contributed to reductions in K_Ca_1.1 activity. Among the eight regulatory β and γ subunits, LRRC26 alone was expressed at high levels in MDA-MB-453 cells and primary and metastatic breast cancer tissues, whereas no significant changes were observed in the expression levels of LRRC26 and activation kinetics of PAX-sensitive outward currents in MDA-MB-453 cells by the treatment with antiandrogens. The treatment with antiandrogens up-regulated the expression of the ubiquitin E3 ligases, FBW7, MDM2, and MDM4 in MDA-MB-453 cells, and the protein degradation of K_Ca_1.1 was significantly inhibited by the respective siRNA-mediated blockade of FBW7 and MDM2. Based on these results, we concluded that K_Ca_1.1 is an androgen-responsive gene in AR-positive breast cancer cells, and its down-regulation through enhancements in its protein degradation by FBW7 and/or MDM2 may contribute, at least in part, to the antiproliferative and antimetastatic effects of antiandrogens in breast cancer cells.

## Introduction

Androgens are steroid hormones that are involved in sexual differentiation and prostate cancer progression in men, and the androgen receptor (AR) is a clinical target in androgen-dependent and castration-resistant prostate cancer (Shafi et al., 2013). More than 500 androgen-responsive genes have been identified from human prostate cancer cells (Nickols and Dervan, 2007; Romanuik et al., 2009). Recent studies showed that AR is widely distributed in primary and metastatic breast cancers, with 60–80% of all, 90% estrogen receptor (ER)-positive, 50% human epidermal growth factor receptor 2 (HER2)-positive, and 75% triple negative (TNBC) breast cancers being AR-positive (Garay and Park, 2012). Furthermore, higher dihydrotestosterone (DHT) levels are associated with a favorable prognosis in AR-positive breast cancer cells (Recchione et al., 1995). Therefore, antiandrogens have potential as a new treatment for breast cancer, preferably TNBC (Gucalp and Traina, 2016; Kono et al., 2017). Among human breast cancer cell lines, MDA-MB-453 cells express AR at high levels, and their proliferation is stimulated by androgens in an AR-dependent manner (Hall et al., 1994). However, limited information is currently available on androgen-responsive genes in breast cancer cells.

The large-conductance Ca^2+^-activated K^+^ channel is composed of a pore-forming α subunit (K_Ca_1.1/KCNMA1) and auxiliary β (KCNMB1-4) and γ [Leucine-Rich Repeat-Containing protein (LRRC) 26, 38, 52, 55] subunits (Latorre et al., 2017). The β and γ subunits modulate macroscopic kinetics and Ca^2+^ and voltage sensitivities. In prostate cancer cells, the γ1 subunit, LRRC26 elicits a large negative shift in the voltage dependence of K_Ca_1.1, thereby activating K_Ca_1.1 at physiological voltages and resting intracellular Ca^2+^ levels (Yan and Aldrich, 2010). Amplified K_Ca_1.1 is associated with a high tumor stage and poor prognosis in breast cancer (Khaitan et al., 2009), and leads to breast cancer proliferation, invasion, and metastasis (Oeggerli et al., 2012; Huang and Jan, 2014). In prostate cancer LNCaP cells, the K_Ca_1.1 gene is amplified by DHT and repressed by antiandrogens (Nickols and Dervan, 2007). The K_Ca_1.1 gene was identified as an androgen-responsive gene in prostate cancer using a long serial analysis of gene expression libraries (LongSAGE) (Romanuik et al., 2009). Our recent study showed the high expression of K_Ca_1.1 in AR-positive MDA-MB-453 cells (Khatun et al., 2016).

Transcriptional, spliceosomal, epigenetic, and proteasomal regulatory pathways play essential roles in the up- and down-regulation of ion channel activity, and the AR signaling pathway cross-talks with its associated molecules such as histone deacetylases (HDACs), microRNAs, and ubiquitin E3 ligases (Ohya et al., 2016; Foot et al., 2017). miR-17-5p regulates the transcription of AR (Gong et al., 2012) and K_Ca_1.1 (Cheng et al., 2016) in cancerous cells. HDAC1, HDAC2, HDAC3, and HDAC6 are highly expressed in breast cancer cells (Seo et al., 2014), and the inhibition of HDAC2 down-regulates the K_Ca_1.1 gene (Khatun et al., 2016). The expression levels of the E3 ubiquitin ligase NEDD4-2 were increased by an androgen treatment in prostate cancer cells (Qi et al., 2003). Additionally, AR is a direct target for the E3 ubiquitin ligase MDM2, which plays an important role in the regulation of the AR signaling pathway (Gaughan et al., 2005). Co-operation between MDM2/FBW7 or MDM2/MDM4 induces the protein degradation of tumor suppressor genes such as p53 and p63 (Galli et al., 2010; Pellegrino et al., 2015).

The aim of the present study is to provide new mechanistic insights into the role of antiandrogens in the repression of K_Ca_1.1 activity in breast cancer cells.

## Materials and methods

### Cell culture

The breast cancer cell lines MDA-MB-453, YMB-1, MCF-7, BT549, and Hs578T were obtained from the RIKEN BioResource Center (RIKEN BRC, Tsukuba, Japan) and Health Science Research Resource Bank (HSRRB, Osaka, Japan). MDA-MB-231 and MDA-MB-468 cells were supplied by Dr. Nishiguchi (Kyoto Pharmaceutical University, Kyoto, Japan). Cells were cultured in Leibovitz's L-15, RPMI 1640, or D-MEM medium (Wako Pure Chemical Industries, Osaka, Japan) supplemented with 10% fetal bovine serum (FBS), 100 units/mL of penicillin/streptomycin, and 2 mM glutamine. Cells were maintained at 37°C in an atmosphere of 5% CO_2_ and 95% air. Commercial FBS contains approximately 0.3 nM testosterone, and the final concentration of testosterone in culture medium is approximately 0.03 nM. Under high testosterone conditions, exogenous DHT (Tokyo Chemical Industry, Tokyo, Japan) was added to the medium at a final concentration of 10 nM. Under testosterone deprivation conditions, charcoal-stripped FBS (Sigma, St. Louis, MO, USA) was added to the medium instead of normal FBS, without the supplementation of 10 nM DHT.

### Cell viability

A cell viability assay using WST-1, which is a colorimetric assay to measure viable cell numbers was performed according to our previous study (Khatun et al., 2016). Briefly, using a density of 10^5^ cells/ml, cells were cultured in duplicate in 96-well plates for 0–5 days. Four hours after the addition of WST-1 reagent (Dojindo, Kumamoto, Japan) into each well, absorbance was measured using a microplate reader Multiskan FC (Thermo Fisher Scientific, Yokohama, Japan) at a test wavelength of 450 nm and a reference wavelength of 620 nm.

### RNA extraction, reverse transcription, and real-time PCR

Total RNA extraction and reverse transcription were performed as previously reported (Khatun et al., 2016). Quantitative real-time PCR was performed using SYBR Green chemistry on an ABI 7500 fast real-time PCR system (Applied Biosystems). Gene-specific PCR primers were designated using Primer Express^TM^ software (Ver 3.0.1, Life Technologies, Carlsbad, CA, USA). The following gene-specific PCR primers of human origin were used for real-time PCR: AR (GenBank accession number: M20132), 2,457–2,583, 127 bp; K_Ca_1.1 (NM_001014797), 1,120–1,239, amplicon = 120 bp; LRRC26 (NM_001013653), 718–837. 120 bp; LRRC38 (NM_001010847), 891–1,022, 130 bp; LRRC52 (NM_001005214), 852–971, 120 bp; LRRC55 (NM_001005210), 553–674, 120 bp; KCNMB1 (NM_004137), 488–608, 121 bp; KCNMB2 (NM_181361), 734–854, 120 bp; KCNMB3 (NM_171828), 556–676, 121 bp; KCNMB4 (NM_014505), 880–1,010, 131 bp; FBW7, (NM_033632), 1,441–1,560, 120 bp; MDM2, (NM_002392), 1,255–1,374, 120 bp; MDM4 (NM_002393), 1,138–1,257, 120 bp; NEDD4-1 (NM_006154), 1,372–1,491, 120 bp; NEDD4-2 (AY312514), 1,039–1,158, 120 bp; β-actin (ACTB) (NM_001101, 411–511), 101 bp. Unknown quantities relative to the standard curve for a particular set of primers were calculated as previously reported (Khatun et al., 2016), yielding the quantitation of gene products relative to ACTB. In order to examine whether the pre-mRNA splicing of K_Ca_1.1 was changed by the treatment with antiandrogens, the PCR amplification of partial fragments including several exons of K_Ca_1.1 was performed as previously reported (Khatun et al., 2016). The amplification profile was as follows: a 15 s denaturation step at 96°C and a 30 s primer extension step at 60°C. The following PCR primers were used: K_Ca_1.1 (NM_001014797) exons 1–4: 104–960, amplicon = 857 bp; exons 5–14: 860–1,849, 990 bp; exons 15–23: 1,761–2,916, 1,156 bp; exons 24–30: 2,688–3,741, 1,054 bp. Amplified products were separated on 1.0% agarose gels, and visualized by ethidium bromide staining. One kbp DNA Ladder One (Nacalai Tesque, Kyoto, Japan) was used as a molecular weight marker.

### Western blotting

Protein lysates were prepared from MDA-MB-453 using RIPA lysis buffer with a protease inhibitor (Thermo Scientific Pierce, Yokohama, Japan) for Western blotting (Khatun et al., 2016). Protein expression levels were measured 48 h after antiandrogen treatments. Equal amounts of protein (20 μg/lane) were subjected to SDS-PAGE (10%). Blots were incubated with anti-AR (110 kDa) (C-19, Santa Cruz Biotechnology), anti-K_Ca_1.1 (100 kDa) (APC-021, Alomone Labs, Jerusalem, Israel), anti-LRRC26 (50 kDa) (ab124181, Abcam, Tokyo Japan), anti-HDAC2 (60 kDa) (H-54, Santa Cruz Biotechnology), and anti-ACTB (43 kDa) (6D1, Medical and Biological Laboratories, Nagoya, Japan) antibodies, then incubated with anti-rabbit or anti-mouse horseradish peroxidase-conjugated IgG (Merck Millipore, Darmstadt, Germany). An enhanced chemiluminescence detection system (GE Healthcare Japan, Tokyo, Japan) was used to detect the bound antibody. The resulting images were analyzed using a VersaDoc5000MP device (Bio-Rad Laboratories, Hercules, CA, USA). The optical density of the protein band signal relative to that of the ACTB signal was calculated using ImageJ software (Ver. 1.42, NIH, USA), and protein expression levels in the vehicle control were then expressed as 1.0.

### Gene silencing by siRNA transfection

Lipofectamine® RNAiMAX reagent (Thermo Fisher Scientific) was used in the siRNA-mediated blockade of AR, FBW7, MDM2, and MDM4 (Life Technologies) (Khatun et al., 2016). Control siRNA (type A) was purchased from Santa Cruz Biotechnology. The expression levels of the target transcripts were assessed 72 h after the transfection of siRNAs using a real-time PCR assay.

### Measurements of K_Ca_1.1 activity by voltage-sensitive dye imaging and whole-cell patch clamp recordings

Membrane potential was measured using the fluorescent voltage-sensitive dye DiBAC_4_(3) as previously reported (Khatun et al., 2016). Briefly, prior to fluorescence measurements, cells were incubated in normal HEPES buffer containing 100 nM DiBAC_4_(3) at room temperature for 20 min, and cells were then continuously incubated in 100 nM DiBAC_4_(3) throughout the experiments. Depolarization responses induced by the selective K_Ca_1.1 blocker, paxilline (PAX, 1 μM) (increase in fluorescence intensity) were measured using an ORCA-Flash2.8 digital camera (Hamamatsu Photonics, Hamamatsu, Japan). Data collection and analyses were performed using an HCImage system (Hamamatsu Photonics). Images were measured every 5 s.

A whole-cell patch clamp was applied to single MDA-MB-453 cells using the HEKA EPC 10 USB amplifier (HEKA Elektronik, Lambrecht/Pfalz, Germany) at room temperature (23 ± 1°C) as previously reported (Khatun et al., 2016). Data acquisition and analyses of whole cell currents were performed using PatchMaster (HEKA). Whole-cell currents were measured in the voltage-clamp mode and induced by 500-ms voltage steps, every 15 s, from−80 mV to +60 mV at a holding potential of −60 mV. The external solution was (in mM): 137 NaCl, 5.9 KCl, 2.2 KCl, 1.2 MgCl_2_, 14 glucose, and 10 HEPES, pH7.4. The pipette solution was (in mM): 140 KCl, 4 MgCl_2_, 3.2 CaCl_2_, 5 EGTA, 10 HEPES, and 2 Na_2_ATP, pH 7.2, with an estimated free Ca^2+^ concentration of 300 nM (pCa 6.5).

To evaluate the voltage-dependence of K_Ca_1.1 channel activation in MDA-MB-453 cells, 30-ms voltage pulses between −80 and +200 mV were applied in 20-mV increments using a holding potential of −80 mV. The whole cell conductance was expressed as the normalized conductance (G/Gmax) calculated from the relative amplitude of the tail currents (deactivation at −60 mV). The relations of normalized conductances (G/Gmax) vs. voltage were fitted by the single-Boltzmann distribution: G/Gmax = 1/(1+exp(V_1/2_-V)/s), where V_1/2_ is the voltage of half-maximal activation and s is the slope factor. The external solution contained (in mM): 140 KCl, 2 MgCl_2_, and 10 HEPES, pH 7.4. The pipette solution was (in mM): 140 KCl, 5 EGTA, and 10 HEPES, pH 7.2.

### Chemicals

Bicalutamide (BCT) was purchased from AdooQ Biosciences (Irvine, CA, USA). Enzalutamide (EZT) was purchased from MedChem Express (Monmouth Junction, NJ, USA). Everolimus, AZD5363, 5,15-diphenylporphyrin (DPP), and Nutlin-3a were purchased from Cayman Chemical (Michigan, MI, USA). SJ172550 was purchased from Tocris Bioscience (Minneapolis, MN, USA). All other reagents were purchased form Sigma-Aldrich (Tokyo, Japan) or Wako Pure Chemicals (Osaka, Japan).

### Statistical analysis

The significance of differences among two and multiple groups was evaluated using the Student's *t*-test and Tukey's test after the F test and ANOVA, respectively. Significance at *p* <0.05 and 0.01 is indicated in the figures. Data are presented as means ± SEM.

## Results

### Inhibition of K_Ca_1.1 activity by the antiandrogen, BCT or EZT in breast cancer cells

**Figure 1:**
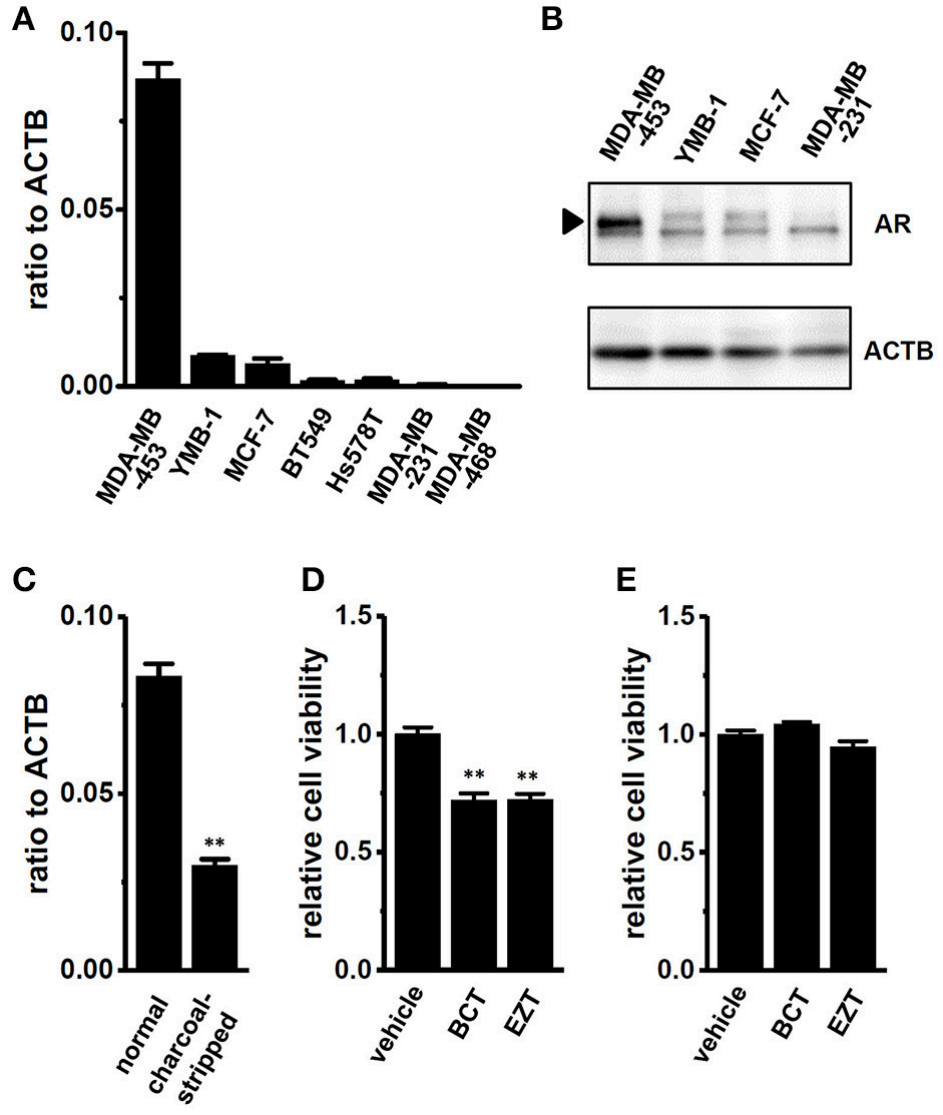
Gene and protein expression of the androgen receptor (AR) in human breast cancer cell lines and effects of antiandrogens on the viability of MDA-MB-453 cells. **(A)** Real-time PCR assay for AR in seven human breast cancer cell lines (*n* = 3 for each). **(B)** Expression of AR proteins (approximately 110 kDa) in MDA-MB-453, YMB-1, MCF-7, and MDA-MB-231 cells. Protein lysates of the examined cells were probed by immunoblotting with anti-AR (upper panel) and anti-ACTB (lower panel) antibodies on the same filter. **(C)** Expression of AR transcripts in MDA-MB-453 cells cultivated for 5 days with normal FBS- and charcoal-stripped FBS-supplemented (10%) medium (*n* = 4 for each). **(D,E)** Effects of treatments with the antiandrogens, bicalutamide (BCT, 1 μM) and enzalutamide (EZT, 1 μM) for 72 h on the viability of MDA-MB-453 cells pre-cultivated for 5 days with normal FCS- **(D)** and charcoal-stripped FCS- **(E)** supplemented medium. The viability of vehicle-treated cells is arbitrarily expressed as 1.0, and data are shown as “relative cell viability” (*n* = 5 for each). Expression levels were expressed as a ratio to ACTB **(A,C)**. Results were expressed as means ± SEM. ^**^p <0.01 vs. the normal FBS group or vehicle control.

We examined the gene and protein expression of AR in several human breast cancer cell lines using real-time PCR and Western blot analyses. As previously reported by Hall et al., the expression levels of AR genes and proteins in MDA-MB-453 cells were markedly higher than those in other cell lines (Figures 1A,B). Cochrane et al. (2014) demonstrated that the proliferation of MDA-MB-453 cells was suppressed by a treatment with the antiandrogen, EZT under an AR stimulation by DHT. In the present study, the antiandrogen, 1 μM BCT or 1 μM EZT significantly suppressed the viability of MDA-MB-453 cells (Figure 1D), and this suppressive effect disappeared by supplementation with charcoal-stripped FBS for 5 days into the culture medium instead of normal FBS (Figure 1E). The significant down-regulation of AR transcripts was detected by the treatment with charcoal-stripped FBS (Figure 1C).

A positive correlation was detected between the expression levels of AR and K_Ca_1.1 transcripts in human primary breast cancer tissues (Supplementary Figure S1A). In the primary breast tumor and corresponding metastatic breast tumor of the same donor (a 65-year-old female, BioChain, Hayward, CA, USA), the expression levels of AR and K_Ca_1.1 transcripts were higher in metastatic tissue than in the primary tumor (Supplementary Figures S3A,B). Our recent study showed that K_Ca_1.1 was the most abundantly expressed in MDA-MB-453 cells, and the pharmacological and siRNA-mediated blockade of K_Ca_1.1 significantly suppressed cell viability (Khatun et al., 2016).

**Figure 2:**
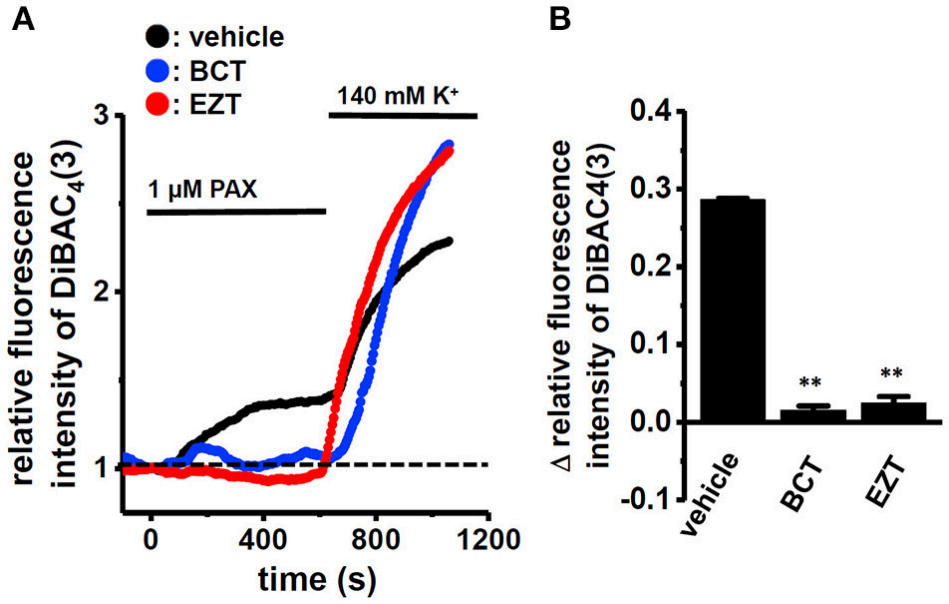
Effects of treatments with antiandrogens for 48 h on 1 μM paxilline (PAX)-induced depolarization responses in MDA-MB-453 cells. **(A)** Measurement of PAX-induced depolarization responses in vehicle (black symbols)-, BCT (blue symbols)-, and EZT (red symbols)-treated MDA-MB-453 cells. The fluorescence intensity of DiBAC_4_(3) before the application of PAX at 0 s is expressed as 1.0. The time courses of changes in the relative fluorescence intensity of DiBAC_4_(3) are shown. **(B)** Summarized data are shown as the PAX-induced Δ relative fluorescence intensity of DiBAC_4_(3) in vehicle-, BCT-, and EZT-treated MDA-MB-453 cells. Cells were obtained from three different batches (59, 60, and 47 cells in each group). Results were expressed as means ± SEM. ^**^p <0.01 vs. the vehicle control.

**Figure 3:**
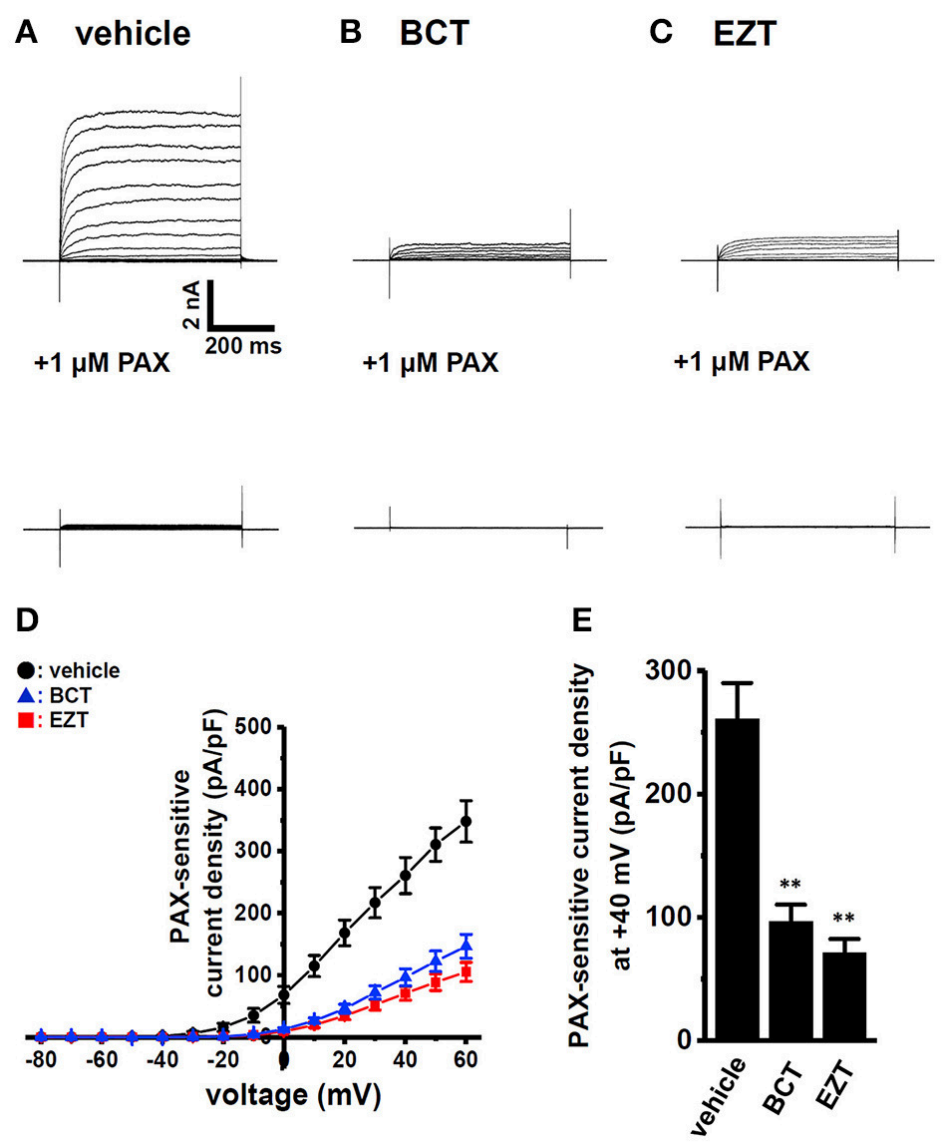
Effects of treatments with antiandrogens for 48 h on paxilline-sensitive outward K^+^ currents in MDA-MB-453 cells. **(A–C)** Currents were elicited by a 500-ms depolarizing voltage step between −80 and +60 mV from a holding potential (−60 mV) with 10-mV increments in MDA-MD-453 cells treated with vehicle (**A**, upper panel), 1 μM BCT (**B**, upper panel), and 1 μM EZT (**C**, upper panel). The application of 1 μM PAX reduced outward currents (**A–C**, lower panel). **(D)** Current density-voltage relationship for PAX-sensitive peak current amplitude. **(E)** Summarized data on PAX-sensitive current density at +40 mV in vehicle- (*n* = 10), BCT- (*n* = 10), and EZT- (*n* = 11) treated MDA-MB-453 cells. Results are expressed as means ± SEM. ^**^p <0.01 vs. the vehicle control.

We then examined the effects of the treatment with 1 μM BCT or 1 μM EZT for 48 h in the presence of DHT on the selective K_Ca_1.1 blocker, PAX (1 μM)-induced depolarization responses in MDA-MB-453 cells using voltage-sensitive fluorescent dye imaging. When fluorescence intensity before the application of PAX was expressed as 1.0, the increase in the relative fluorescence DiBAC_4_(3) intensity induced by PAX was markedly smaller in BCT- or EZT-treated MDA-MB-453 cells than in the vehicle control (Figure 2A). Ten minutes after the application of PAX, 140 mM K^+^ solution was applied to cells in order to omit dead cells and insufficiently dye-loaded cells. Summarized data showed that the change in relative fluorescence intensity [Δ relative fluorescence intensity of DiBAC_4_(3)] was significantly lower in BCT- or EZT-treated MDA-MB-453 cells than in the vehicle control (Figure 2B). No significant differences of high K^+^-induced depolarization responses were found among three groups (not shown). As shown in Figures 1A,B YMB-1 and MDA-MB-231 cells expressed K_Ca_1.1 at low levels. Significant PAX-induced depolarization responses were not observed in these cells (Supplementary Figure S1B). Similar results were obtained using whole-cell patch clamp recording. Currents were elicited by a 500-ms depolarizing voltage step between −80 mV and +60 mV from a holding potential (−60 mV) with 10-mV increments. Figure 3A upper panel shows typical current traces recorded from MDA-MB-453 cells treated with 0.1% DMSO (vehicle control) for 48 h. PAX almost completely suppressed outward currents (Figure 3A, lower panel). As shown in Figures 3B,C (upper panel), outward currents were largely inhibited by the treatment with BCT or EZT for 48 h. In Figure 3D, the peak amplitudes of PAX-sensitive outward currents were plotted against test potentials as the current density after normalization by cell capacitance (pA/pF). The peak K_Ca_1.1 current density at +40 mV was suppressed by more than 60% by the treatment with BCT (*n* = 10) or EZT (*n* = 11) [*p* <0.01 vs. vehicle control (*n* = 10)] (Figure 3E).

### Decreased gene and protein expression levels of K_Ca_1.1 by antiandrogens in breast cancer cells

**Figure 4:**
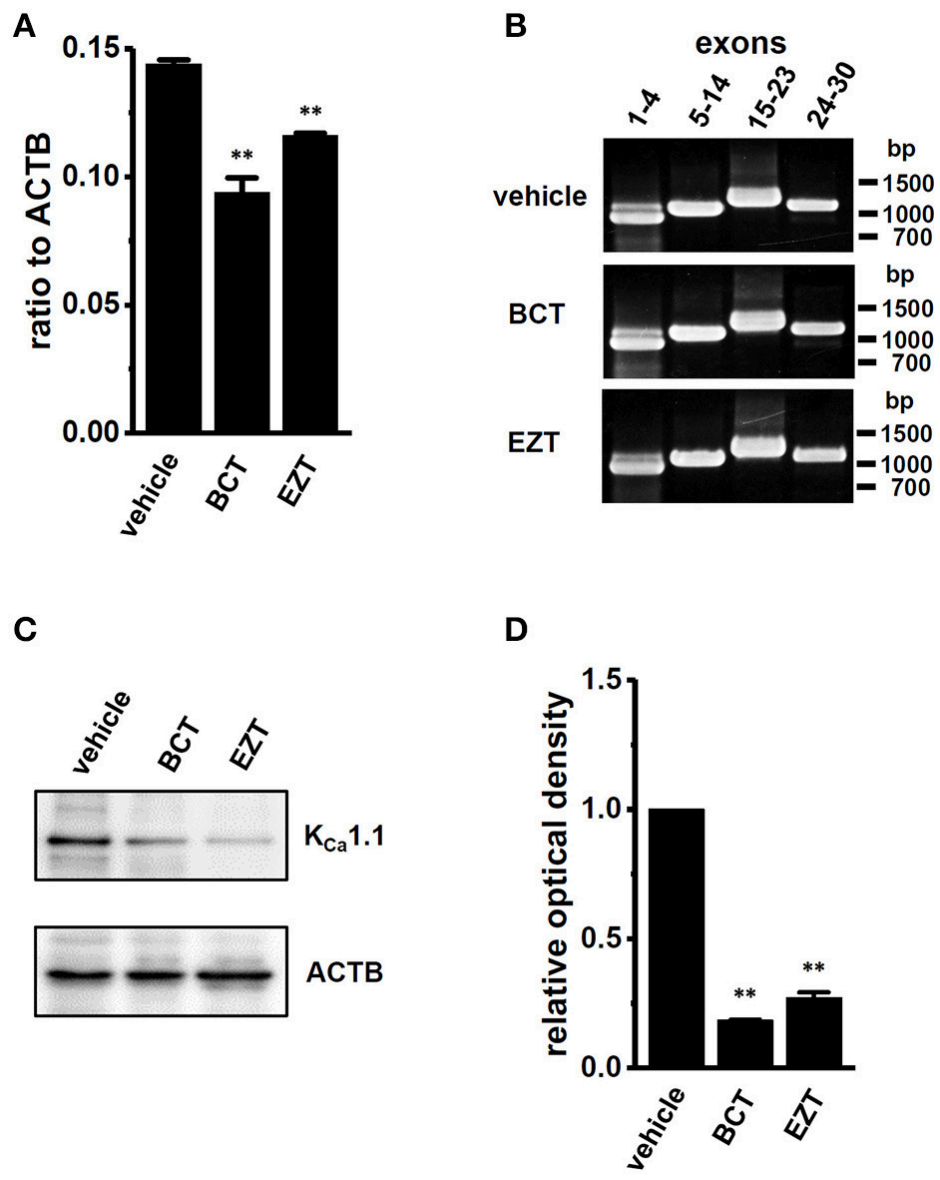
Down-regulation of K_Ca_1.1 transcripts and proteins in MDA-MB-453 cells by treatments with antiandrogens for 48 h. **(A)** Real-time PCR assay for K_Ca_1.1 in vehicle-, 1 μM BCT-, and 1 μM EZT-treated MDA-MB-453 cells (*n* = 4 for each). Expression levels were expressed as a ratio to ACTB. **(B)** Band patterns on agarose gels for the PCR products of K_Ca_1.1 exons (exons 1–4, 5–14, 15–23, and 24–30) in vehicle-, 1 μM BCT-, and 1 μM EZT-treated MDA-MB-453 cells. A DNA molecular weight marker is indicated on the right of the gel. **(C)** Protein lysates of vehicle-, 1 μM BCT-, and 1 μM EZT-treated MDA-MB-453 cells were probed by immunoblotting with anti-K_Ca_1.1 (upper panel) and anti-ACTB (lower panel) antibodies on the same filter. **(D)** Summarized results were obtained as the optical density of K_Ca_1.1 and ACTB band signals. After compensation for the optical density of the K_Ca_1.1 protein band signal with that of the ACTB signal, the K_Ca_1.1 signal in the vehicle control was expressed as 1.0 (*n* = 3 for each). Results are expressed as means ± SEM. ^**^p <0.01 vs. the vehicle control.

Quantitative real-time PCR examinations were performed in order to investigate the effects of antiandrogens on the expression levels of K_Ca_1.1 transcripts. As shown in Figure 4A, the expression levels of K_Ca_1.1 transcripts in MDA-MB-453 cells treated with 1 μM BCT or 1 μM EZT for 48 h were significantly lower than those in the vehicle control (*n* = 4 for each, *p* <0.01); however, inhibitory rates (20–30%) were largely dissociated with those of K_Ca_1.1 activity in Figures 2B, 3E. Similarly, the siRNA-mediated inhibition of AR expression resulted in the down-regulation of K_Ca_1.1 transcripts (Supplementary Figures S1C,D). Human K_Ca_1.1 encoded by the KCNMA1 gene is composed of 30 exons on chromosome 10q22, and more than 20 alternatively spliced variants have been identified in the N- and C-termini (Mahmoud and McCobb, 2004). In addition, Bell et al. (2010) reported that the stress axis-regulated exon (STREX) splicing of K_Ca_1.1 was regulated by testosterone in the pituitary. Similar to our previous study (Khatun et al., 2016), non-quantitative RT-PCR examinations were performed using specific primers for exons 1–4, exons 5–14, exons 15–23, and exons 24–30. As shown in Figure 4B, the band patterns on agarose gels in the BCT (middle panel)- and EZT (lower panel)-treated groups were the same as those in the vehicle (upper panel)-treated group, suggesting that antiandrogens do not affect any pre-mRNA splicing processes of K_Ca_1.1 in MDA-MB-453 cells. The protein expression levels of K_Ca_1.1 were subsequently assessed by Western blotting. The treatment of MDA-MB-453 cells with antiandrogens resulted in a greater than 70% decrease in the expression levels of the K_Ca_1.1 protein (*n* = 4 for each, *P* <0.01 vs. vehicle control) (Figures 4C,D), which is consistent with the inhibitory rates of K_Ca_1.1 activity by antiandrogens shown in Figures 2B, 3E. These results suggest that the antiandrogen-induced inhibition of K_Ca_1.1 activity is mainly due to the promotion of K_Ca_1.1 protein degradation.

### Effects of antiandrogens on expression levels of K_Ca_1.1 regulatory subunits and K_Ca_1.1 activation kinetic in breast cancer cells

**Figure 5:**
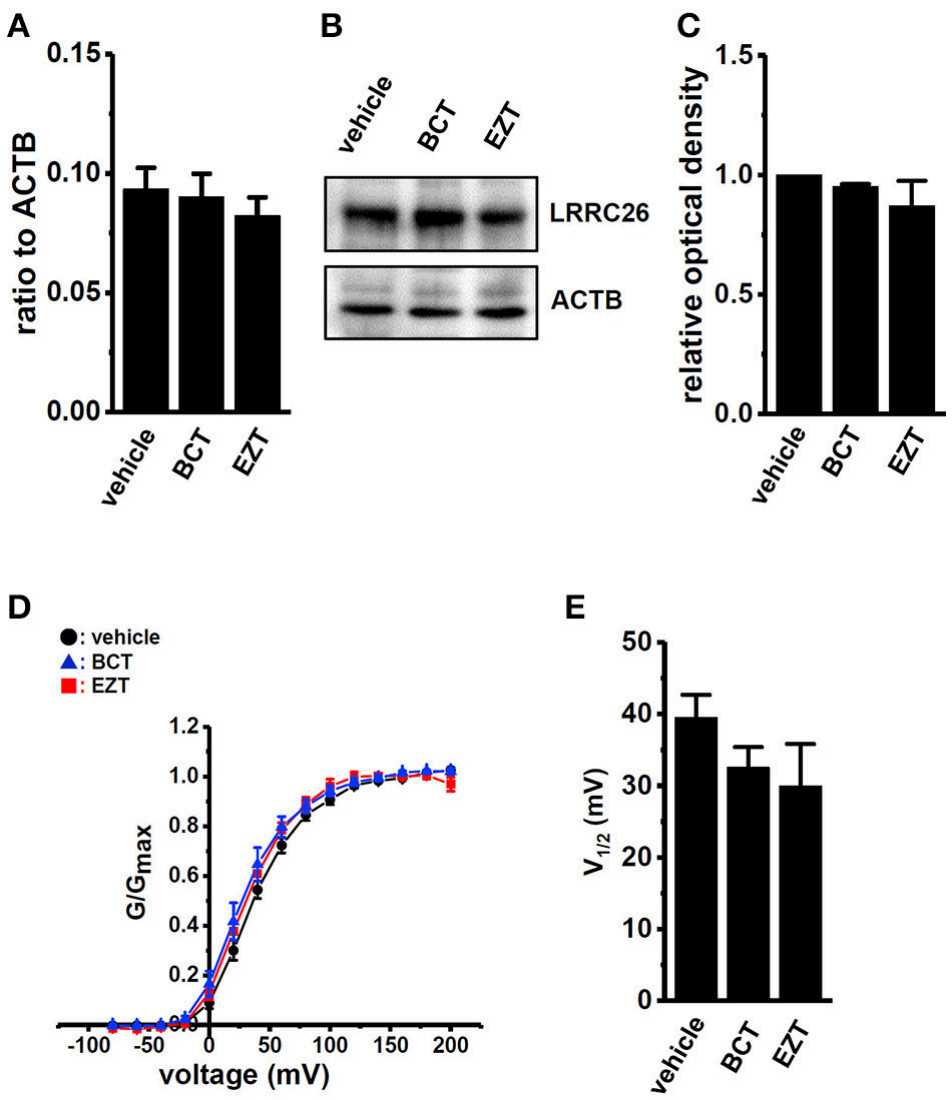
Effects of treatments with antiandrogens on expression levels of the K_Ca_1.1 regulatory γ subunit, LRRC26 transcripts and proteins and on the voltage dependency of K_Ca_1.1 currents in MDA-MB-453 cells. **(A)** Real-time PCR assay for LRRC26 in vehicle-, 1 μM BCT-, and 1 μM EZT-treated MDA-MB-453 cells (*n* = 4 for each). Expression levels were expressed as a ratio to ACTB. **(B)** Protein lysates of vehicle-, 1 μM BCT-, and 1 μM EZT-treated MDA-MB-453 cells were probed by immunoblotting with anti-LRRC26 (upper panel) and anti-ACTB (lower panel) antibodies on the same filter. **(C)** Summarized results were obtained as the optical density of LRRC26 and ACTB band signals. After compensation for the optical density of the LRRC26 protein band signal with that of the ACTB signal, the LRRC26 signal in the vehicle control was expressed as 1.0 (*n* = 4 for each).**(D)** The voltage dependency of activation curves was derived from the current (I)-voltage (V) relationship in vehicle- (*n* = 13), BCT- (*n* = 9), and EZT- (*n* = 9) treated MDA-MB-453 cells. I–V curves were obtained using the protocol with pulses to potentials ranging between −80 mV and +200 mV for 30 ms, followed by a voltage step to −60 mV for 100 ms. The peak current measured at each potential was divided by (V-V_rev_), where V is the test potential and V_rev_ is the reversal potential. Conductance was then normalized and fit to a standard Boltzmann equation. **(E)** Summarized data of the half-maximal voltage (V_1/2_) of activation. Results were expressed as means ± SEM.

Real-time PCR examinations were performed in order to investigate the effects of antiandrogens on the transcriptional expression levels of four K_Ca_1.1 β subunits (KCNMB1-4) and four K_Ca_1.1 γ subunits (LRRC26, 38, 52, and 55). Similar to K_Ca_1.1-expressing prostate cancer cells (Yan and Aldrich, 2010), LRRC26 was the main auxiliary subunit of K_Ca_1.1 in K_Ca_1.1-expressing breast cancer MDA-MB-453 cells (Figure 5A, vehicle) and breast cancer tissues (Supplementary Figure S3C). The other seven subunits were less abundantly expressed in MDA-MB-453 cells (less than 0.05 in arbitrary units; Supplementary Figure S2). The expression levels of LRRC26 transcripts in metastatic breast cancer tissue were significantly higher than those in the primary tumor (Supplementary Figure S3C). We then examined the effects of antiandrogens on the expression levels of LRRC26 transcripts and proteins in MDA-MB-453 cells, and found no significant changes following the treatment with antiandrogens (Figures 5A–C). We further assessed the half-activation voltage (V_1/2_) of PAX-sensitive outward currents in the virtual absence of [Ca^2+^]_i_ using whole-cell patch clamp recording. Consistent with the above results, no significant differences in V_1/2_ by the antiandrogen treatment were found in MDA-MB-453 cells (Figures 5D,E). Both antiandrogens caused a slightly hyperpolarizing shift in the voltage of V_1/2_: vehicle control, 39.5 ± 3.2 (*n* = 13); BCT-treated, 32.5 ± 2.8 (*n* = 9); EZT-treated, 30.0 ± 5.9 (*n* = 9), *p* >0.05 vs. vehicle control.

### Involvement of the PI3K/Akt/mTOR signaling pathway in the down-regulation of K_Ca_1.1 transcripts by the antiandrogen treatment

**Figure 6:**
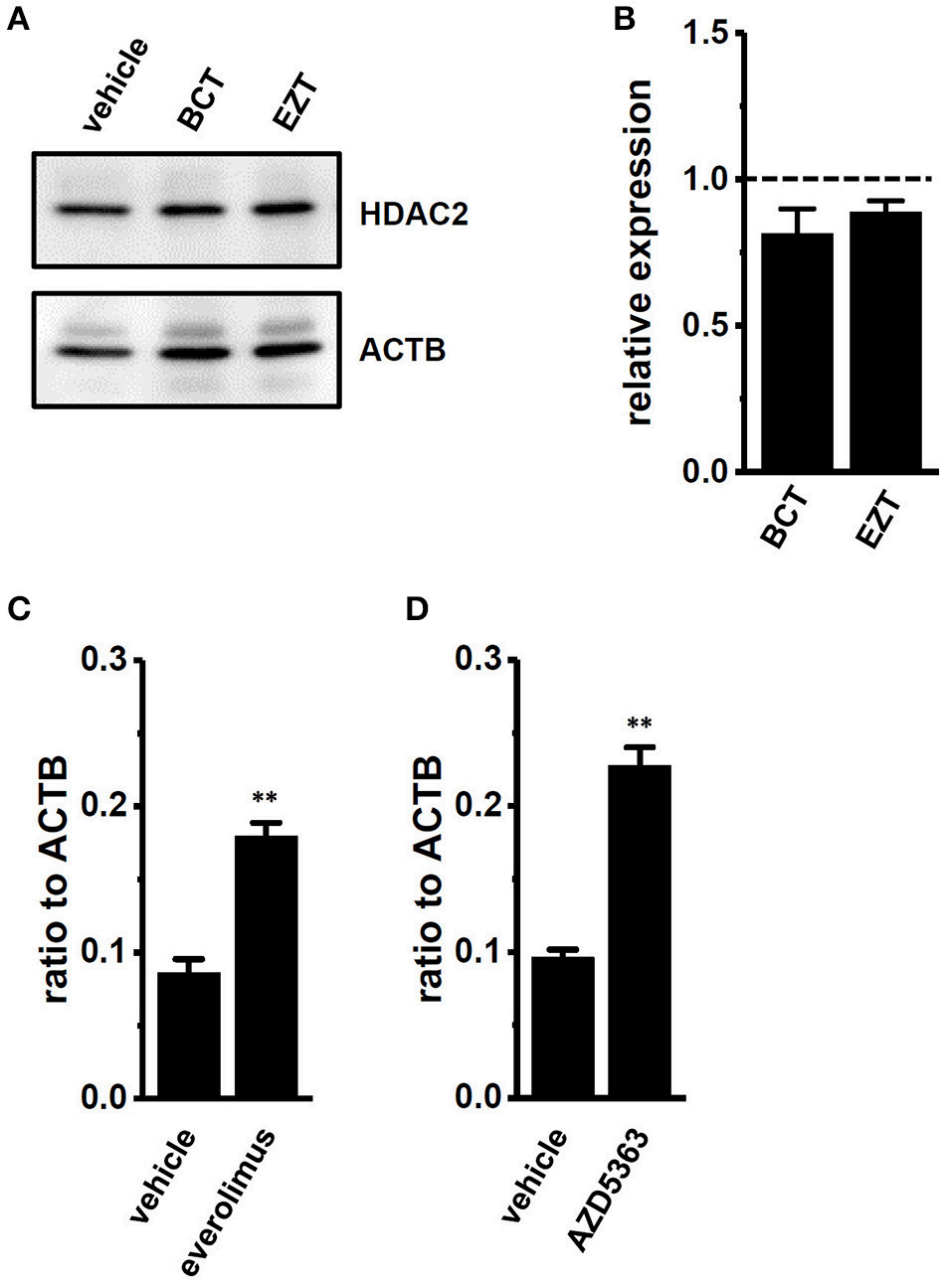
Effects of treatments with antiandrogens on expression levels of histone deacetylase (HDAC) 2 proteins and effects of mTOR and AKT inhibitors on expression levels of K_Ca_1.1 transcripts in MDA-MB-453 cells. **(A)** Protein lysates of vehicle-, 1 μM BCT-, and 1 μM EZT-treated MDA-MB-453 cells were probed by immunoblotting with anti-HDAC2 (upper panel) and anti-ACTB (lower panel) antibodies on the same filter. **(B)** Summarized results were obtained as the optical density of HDAC2 and ACTB band signals. After compensation for the optical density of the HDAC2 protein band signal with that of the ACTB signal, the HDAC2 signal in the vehicle control was expressed as 1.0 (dotted line, *n* = 4 for each). **(C,D)** Real-time PCR assay for K_Ca_1.1 in vehicle-, 10 nM everolimus- **(C)**, and 1 μM AZD5363 **(D)**-treated MDA-MB-453 cells (*n* = 4 for each). Expression levels were expressed as a ratio to ACTB. Results are expressed as means ± SEM. ^**^p <0.01 vs. the vehicle control.

We recently demonstrated that K_Ca_1.1 transcripts were down-regulated by histone deacetylase (HDAC) 2 inhibition, and that the active vitamin D-induced protein degradation of HDAC2 was involved in the down-regulation and reduced activity of K_Ca_1.1 (Khatun et al., 2016). We examined the effects of antiandrogens on HDAC2 protein expression, and found no significant differences between the treatment with vehicle and BCT or EZT in MDA-MB-453 cells (Figures 6A,B).

The PI3K/AKT/mTOR signaling pathway includes important transcriptional regulators in cancer cells. mTOR is the downstream gene that is positively regulated by AR in prostate cancer cells, and the repression of the mTOR signal also exhibits a compensatory increase in AR function (Wu et al., 2010). In breast cancer MDA-MB-453 cells, the treatment with either the mTOR inhibitor, everolimus (10 nM) or the Akt inhibitor, AZD5363 (1 μM) significantly up-regulated K_Ca_1.1 transcription (Figures 6C,D). These results suggest that the PI3K/AKT/mTOR signaling pathway may be involved in the transcriptional repression of K_Ca_1.1 by antiandrogens in breast cancer cells. AKT/mTOR inhibitors suppressed the viability of MDA-MB-453 cells (Supplementary Figures S4A,B) and up-regulated AR transcription (Supplementary Figures S4C,D).

### Involvement of E3 ubiquitin ligases in the protein degradation of K_Ca_1.1 by antiandrogens in breast cancer cells

**Figure 7:**
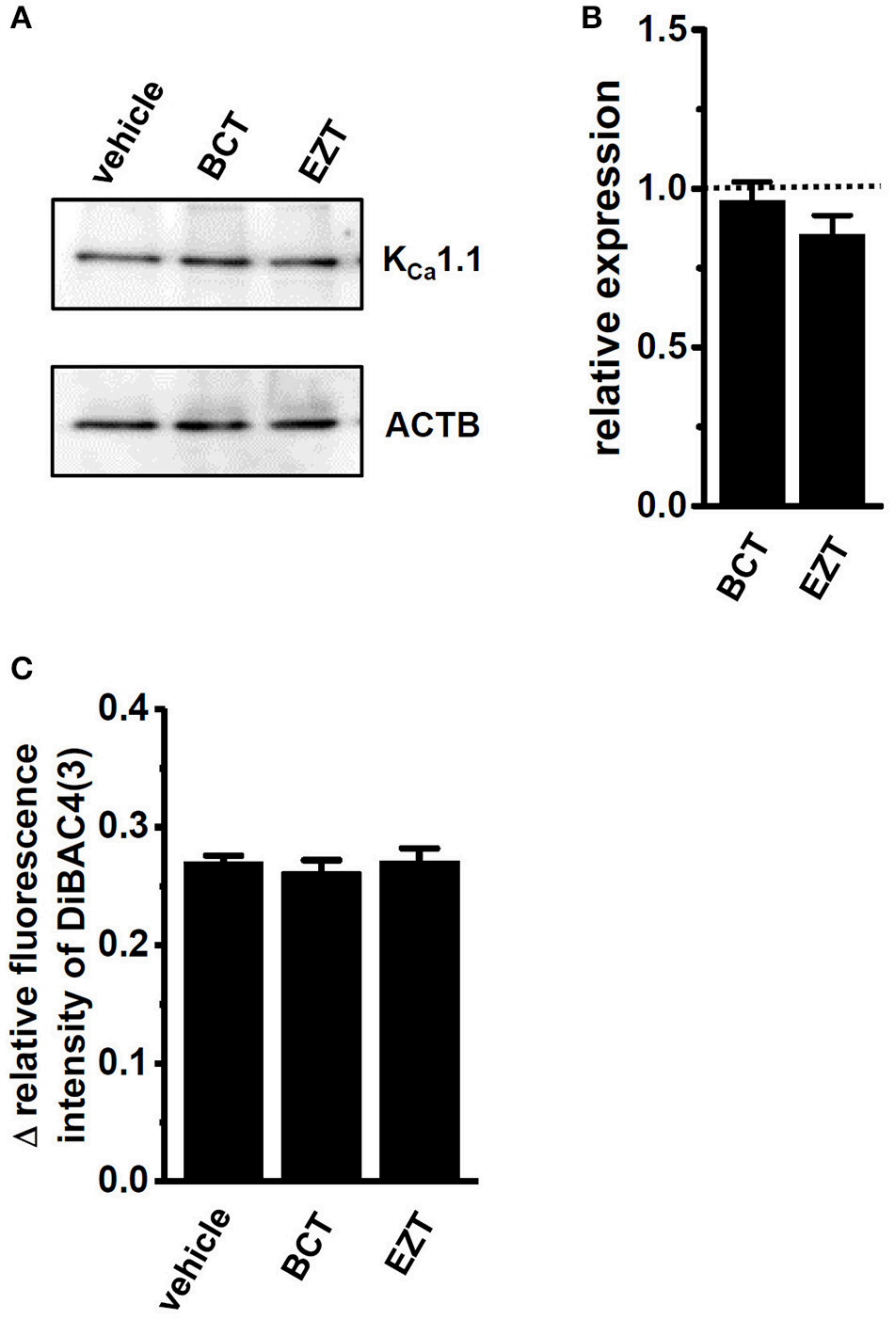
Effects of the potent proteasome inhibitor, MG132 (100 nM) in the presence of 10 nM DHT on expression levels of K_Ca_1.1 proteins in antiandrogen-treated MDA-MB-453 cells. MG132 was applied 24 h after the treatment with antiandrogens. **(A)** Protein lysates of vehicle-, 1 μM BCT-, and 1 μM EZT-treated MDA-MB-453 cells were probed by immunoblotting with anti-K_Ca_1.1 (upper panel) and anti-ACTB (lower panel) antibodies on the same filter. **(B)** Summarized results were obtained as the optical density of K_Ca_1.1 and ACTB band signals. After compensation for the optical density of the K_Ca_1.1 protein band signal with that of the ACTB signal, the K_Ca_1.1 signal in the vehicle control was expressed as 1.0 (dotted line, *n* = 3 for each). **(C)** Measurement of PAX-induced depolarization responses in vehicle-, BCT-, and EZT-treated MDA-MB-453 cells. Summarized data are shown as the PAX-induced Δ relative fluorescence intensity of DiBAC_4_(3) in vehicle-, BCT-, and EZT-treated MDA-MB-453 cells. Cells were obtained from three different batches (64, 43, and 35 cells in each group). Results were expressed as means ± SEM.

As described above, the antiandrogen-induced inhibition of K_Ca_1.1 activity appears to be mainly due to the protein degradation of K_Ca_1.1. In order to elucidate the involvement of protein degradation processes in the antiandrogen-induced down-regulation of K_Ca_1.1 proteins in MDA-MB-453 cells, the effects of the potent proteasome inhibitor, MG132 (100 nM) on antiandrogen-induced K_Ca_1.1 protein degradation were examined. MG132 was added 24 h after the treatment with antiandrogens. Reductions in K_Ca_1.1 proteins induced by the treatment with BCT or EZT were almost completely prevented by the MG132 treatment for 24 h (Figures 7A,B). Consistently, the significant attenuation of PAX-induced depolarization responses in BCT- or EZT-treated MDA-MB-453 cells almost completely disappeared after the MG132 treatment (Figure 7C).

**Figure 8:**
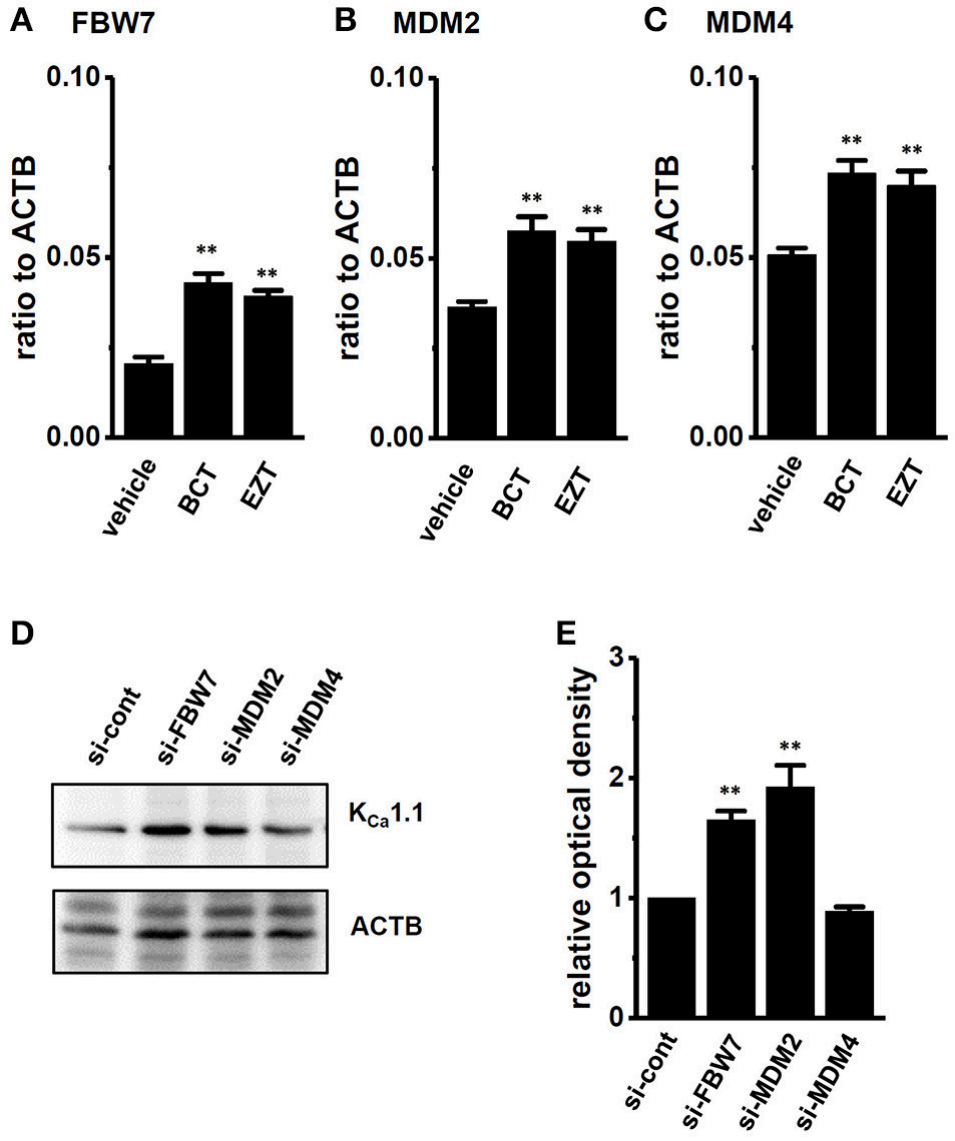
Effects of treatments with antiandrogens on expression levels of ubiquitin E3 ligases, FBW7, MDM2, and MDM4 transcripts and effects of the siRNA-mediated inhibition of ubiquitin E3 ligases on expression levels of K_Ca_1.1 proteins in MDA-MB-453 cells. **(A–C)** Real-time PCR assay for FBW7 **(A)**, MDM2 **(B)**, and MDM4 **(C)** in vehicle-, 1 μM BCT-, and 1 μM EZT-treated MDA-MB-453 cells (*n* = 4 for each). **(D)** Protein lysates of FBW7, MDM2, and MDM4 siRNA-transfected MDA-MB-453 cells (for 72 h) were probed by immunoblotting with anti-K_Ca_1.1 (upper panel) and anti-ACTB (lower panel) antibodies on the same filter. **(E)** Summarized results were obtained as the optical density of K_Ca_1.1 and ACTB band signals. After compensation for the optical density of the K_Ca_1.1 protein band signal with that of the ACTB signal, the K_Ca_1.1 signal in the vehicle control was expressed as 1.0 (*n* = 4 for each). Expression levels were expressed as a ratio to ACTB. Results are expressed as means ± SEM. ^**^p <0.01 vs. the vehicle control or control siRNA (si-cont).

We then identified the ubiquitin E3 ligase(s) involved in the antiandrogen-induced protein degradation of K_Ca_1.1 in MDA-MB-453 cells. MDM2 is the ubiquitin E3 ligase regulating AR-downstream target genes by AR degradation (Qi et al., 2003). The complexes, MDM2/MDM4 (MDMX) and MDM2/FBW7 (F-box/WD repeat-containing protein 7) promote protein ubiquitination and degradation (Gaughan et al., 2005; Galli et al., 2010). Moreover, NEDD4s (neural precursor cell expressed developmentally down-regulated protein 4) regulates ion channel functions via their ubiquitination (Qi et al., 2003; Foot et al., 2017). We examined changes in the expression levels of FBW7, MDM2, MDM4, NEDD4-1, and NEDD4-2 transcripts by the antiandrogen treatment for 48 h. As shown in Figures 8A–C, the expression of FBW7, MDM2, and MDM4 was significantly up-regulated by the treatment with BCT or EZT, without changes in the expression levels of NEDD4-1 and NEDD4-2 (Supplementary Figure S5). The significant up-regulation of FBW7, MDM2, and MDM4 occurred 24 h after the antiandrogen treatment (Supplementary Figure S6). We then examined the effects of the siRNA-mediated inhibition of FBW7, MDM2, and MDM4 on K_Ca_1.1 protein expression in MDA-MB-453 cells by Western blotting. As shown in Figures 8D,E a significant increase in the protein expression levels of K_Ca_1.1 was observed in FBW7- and MDM2-down-regulated MDA-MB-453 cells; however, no significant changes were observed in MDM4-down-regulated cells. Approximately 50–70% of transcripts were inhibited by siRNA transfection (Supplementary Figure S7). The antiandrogen-induced protein degradation of K_Ca_1.1 was observed by the treatment with the MDM4 inhibitor, SJ172550 (20 μM) for 12 h (Supplementary Figure S8B); however, it disappeared following the addition of the MDM2 inhibitor, nutrin-3a (10 μM) (Supplementary Figures S8A). Similarly, the inhibition of PAX-induced depolarization responses by antiandrogens disappeared with the treatment with nutrin-3a, but not SJ172550 (Supplementary Figures S8C,D). Consistent with the above results, the up-regulation of the ubiquitin E3 ligases FBW7 and MDM2 may contribute to the antiandrogen-induced protein degradation of K_Ca_1.1 in MDA-MB-453 cells.

## Discussion

The large-conductance Ca^2+^-activated K^+^ channel K_Ca_1.1 encoded by the KCNMA1 gene is overexpressed in breast cancer cells, and plays a crucial role in breast cancer proliferation, invasion, and metastasis (Khaitan et al., 2009; Huang and Jan, 2014). AR is expressed in most breast cancers and has been proposed as a therapeutic target for triple negative breast cancer and breast cancer with drug resistance (Gucalp and Traina, 2016; Kono et al., 2017). Lehmann et al. (2011) categorized into six TNBC groups based on significant heterogeneity. The luminal AR (LAR) subtype was characterized by high expression of the AR, AR downstream effectors, and so on. The AR-positive breast cancer MDA-MB-453 cells have a similar molecular profiling to LAR subtype. However, the regulatory mechanisms of K_Ca_1.1 expression through AR signaling are poorly understood. We herein demonstrated 1) the prevention of K_Ca_1.1 activity by a long-term treatment with antiandrogens in MDA-MB-453 cells (Figures 2,3), 2) the antiandrogen-induced transcriptional repression of K_Ca_1.1 in MDA-MB-453 cells (Figure 4A), and 3) the antiandrogen-induced enhancement of K_Ca_1.1 protein degradation mediating FBW7 and/or MDM2 in MDA-MB-453 cells (Figures 4C,D, 7, 8).

Recent studies reported acute testosterone-induced, non-genomic K_Ca_1.1 activation in vascular and urinary bladder smooth muscle cells (Hristov et al., 2016; Ruamyod et al., 2017); however, the long-term effects of an AR stimulation or inhibition on K_Ca_1.1 expression remain inconclusive. As shown in Figures 2, 3 K_Ca_1.1 activity measured by voltage-sensitive dye imaging and whole-cell patch clamp recording was significantly prevented by the antiandrogen treatment in MDA-MB-453 cells. Concomitant with the antiandrogen-induced inhibition of K_Ca_1.1 activity, significant decreases were observed in the expression levels of K_Ca_1.1 transcripts and proteins by the antiandrogen treatment (Figure 4). The expression levels of K_Ca_1.1 proteins were markedly lower (more than 70%) following the antiandrogen treatment than those of K_Ca_1.1 transcripts (20-30%). Therefore, the main mechanistic cause of antiandrogen actions in the inhibition of K_Ca_1.1 activity in breast cancer cells is considered to be the enhancement of K_Ca_1.1 protein degradation. In prostate cancer cells, K_Ca_1.1 amplification is observed in the early stages of carcinogenesis (Ohya et al., 2009; Altintas et al., 2013); however, K_Ca_1.1 was more strongly expressed in metastatic breast cancer tissues than in the primary tumor (Supplementary Figure S3B).

On the other hand, the auxiliary γ 1 subunit, LRRC26, which activates K_Ca_1.1 via a large negative shift in voltage dependence, is mainly expressed in MDA-MB-453 cells and breast cancer tissues (Supplementary Figures S2, S3). No significant changes in the expression levels of LRRC 26 transcripts and proteins were found in antiandrogen-treated MDA-MB-453 cells (Figures 5A–C). As shown in Figures 5D,E a slight shift in the half maximal voltage (V_1/2_) in a negative direction was noted. This may have been due to a relative increase in LRRC26 to K_Ca_1.1 in the plasma membrane by the down-regulation of K_Ca_1.1. In line with the above results, antiandrogen-induced K_Ca_1.1 protein degradation may be, at least in part, responsible for reduced viability in MDA-MB-453 cells.

Our previous study demonstrated that the protein degradation of type 2 histone deacetylase (HDAC2) by vitamin D receptor (VDR) agonists contributed to the transcriptional repression of K_Ca_1.1 in MDA-MB-453 cells (Khatun et al., 2016). However, no significant changes in the protein expression levels of HDAC2 were found in antiandrogen-treated MDA-MB-453 cells (Figures 7A,B). These results are reasonable because HDACs are upstream effectors of the AR signaling pathway in prostate cancer (Welsbie et al., 2009).

MicroRNAs control gene expression post-transcriptionally by preventing the protein degradation of target RNAs. Östling et al. (2011) identified 71 unique miRNAs that influenced AR levels in prostate cancer cells (Östling et al., 2011), and previous studies identified several K_Ca_1.1-down-regulating miRNAs (Östling et al., 2011; Tatro et al., 2013; Kroiss et al., 2015; Cheng et al., 2016; Samuel et al., 2016; Lu et al., 2017). We identified miR-9, miR-17-5p, miR-135a, and miR-449a as androgen-regulated, K_Ca_1.1-down-regulating miRNAs in prostate cancer. Shi et al. recently reported miRNAs associated with AR expression in breast cancer cell lines, excluding MDA-MB-453 cells (Shi et al., 2017); however, no common miRNAs regulated by AR were found. Further studies are required in order to identify the miRNAs, including miR-9, miR-17-5p, miR-135a, and miR-449a, that contribute to K_Ca_1.1 translational repression via mRNA degradation in AR-overexpressing breast cancer tissues. A recent study showed that miR-17-5p also down-regulates K_Ca_1.1 in another malignant tissue, the pleural mesothelioma (Cheng et al., 2016). In breast cancer cells, miR-17-5p negatively regulates the expression of phosphatase and tensin homolog, PTEN (Li et al., 2017), and the activation of PTEN inhibits the PI3K/AKT/mTOR signaling pathway (Guo et al., 2015), suggesting that the overexpression of miR-17-5p may promote the PI3K/AKT/mTOR signaling pathway. Consistent with these studies, the overexpression of the other AR-responsive miRNAs, miR-135a and miR-9 increased the phosphorylation of AKT (Zheng et al., 2016) and suppressed PTEN expression in carcinoma cells (Lu et al., 2016). In contrast, the overexpression of miR-449a suppressed AKT activation in hepatocellular carcinoma (Chen et al., 2015). The present study showed that AKT/mTOR inhibitors significantly up-regulated the expression levels of K_Ca_1.1 transcripts (Figures 6C,D). Therefore, the activation of the PI3K/AKT/mTOR signaling pathway through the down-regulation of PTEN may be involved in the antiandrogen-induced transcriptional repression of K_Ca_1.1 in MDA-MB-453 cells. The STAT3 signaling pathway is also important in the regulation of breast cancer proliferation and apoptosis. STAT3 enhances the expression of the downstream target genes of AR signaling via the transactivation of AR in prostate cancer cells (De Miguel et al., 2003; Östling et al., 2011), indicating that STAT3 is the upstream signal of AR. A treatment with the STAT3 inhibitor, 5,15-DPP (10 μM) significantly down-regulated AR and K_Ca_1.1 transcription (Supplementary Figures S9).

A number of ubiquitin ligases are known to regulate ion channel functions, and the ubiquitin-regulated down-regulation of ion channels has been strongly implicated in human pathologies including cancers (Foot et al., 2017). As shown in Figure 7, the proteasome inhibitor MG132 almost completely suppressed the inhibitory effects of antiandrogens on K_Ca_1.1 protein expression in MDA-MB-453 cells. The ubiquitin ligase, NEDD4-2 (also refer to NEDD4L) is responsible for the protein degradation of voltage-gated K^+^ channels (K_V_1.3 and K_V_11.1), resulting in the prevention of their activities (Kang et al., 2015; Vélez et al., 2016). However, Qi et al. (2003) reported that NEDD4-2 transcription was “enhanced” by an AR stimulation in prostate cancer, suggesting that an antiandrogen treatment “decreases” protein degradation via NEDD4-2. As shown in Supplementary Figures S5B, no significant changes were found in the expression levels of NEDD4-2 transcripts in antiandrogen-treated MDA-MB-453 cells.

AR is a direct target for MDM2-mediated ubiquitylation in prostate cancer cells (Gaughan et al., 2005) Of interest, MDM2 activation by phosphorylation is negatively regulated by the downstream target genes of AR signaling (Ogawara et al., 2002). Additionally, MDM2/FBW7 and MDM2/MDM4 co-operate to induce the protein degradation of tumor suppressor genes (Galli et al., 2010; Pellegrino et al., 2015). As shown in Figures 8A–C, FBW7, MDM2, and MDM4 transcripts were up-regulated in antiandrogen-treated MDA-MB-453 cells. This is the first study to show that FBW7/MDM2/MDM4 transcription is regulated by AR signaling. The androgen-dependent regulation of miRNA expression may be involved in FBW7/MDM2/MDM4 mRNA degradation. Further studies are needed in order to identify mechanistic target(s). Of importance, their siRNA-mediated inhibition (Figures 8D,E) and pharmacological blockade (Supplementary Figure S8) strongly indicated that MDM2 and FBW7 both contributed to the antiandrogen-induced promotion of K_Ca_1.1 protein degradation. Previous studies showed that the phosphorylation of FBW7 and MDM2 via PI3K and AKT modulates the protein degradation of their targets (Ogawara et al., 2002; Schülein et al., 2011). Furthermore, phosphorylated MDM2 prevents PTEN expression via p53 degradation (Mayo et al., 2002). These insights into the AR signaling pathway will provide novel mechanisms for functional K_Ca_1.1 regulation in breast cancer cells.

Our previous study also showed the enhanced protein degradation of K_Ca_1.1 by a treatment with VDR agonists (Khatun et al., 2016). Significant increases in the expression levels of MDM2 and MDM4 transcripts were also found in VDR agonist (calcitriol and calcipotriol)-treated MDA-MB-453 cells (not shown). Since the inhibition of AR stimulates VDR levels (Mooso et al., 2010), antiandrogens and VDR agonists may use common inhibitory mechanisms on K_Ca_1.1 activity.

In conclusion, the present results suggest that the ubiquitin E3 ligases FBW7 and MDM2 in the downstream of AR signaling play an important role in K_Ca_1.1 protein degradation processes in breast cancer cells and provide novel insights into AR, VDR, and ubiquitin E3 kinase-targeted therapy for breast cancer with a poor prognosis. In this study, we focused on the long-term effect of antiandrogens on K_Ca_1.1 expression and the underlying mechanisms in breast cancer MDA-MB-453 cells. In order to understand the ionic mechanisms underlying antiandrogen-induced inhibition of breast cancer cell proliferation and migration, further studies will be needed to clarify genomic and non-genomic regulation of the other ion channels by antiandrogens. Indeed, it has been recently reported that the K^+^ channels such as voltage-gated K_V_11.1, and Ca^2+^-activated K_Ca_3.1 contribute to breast cancer cell development (Fukushiro-Lopes et al., 2017; Steudel et al., 2017).

## Author contributions

AK and SO participated in research design. AK, MS, HK, MK, MayF, MR, SN, and SO conducted the experiments. AK, MS, HK, MK MayF, MR, JK, SN, MasF, and SO performed data analyses. AK, HK, and SO contributed to the writing of the manuscript.

### Conflict of interest statement

The authors declare that the research was conducted in the absence of any commercial or financial relationships that could be construed as a potential conflict of interest.
