# Supplementary material for: Transcriptional Repression and Protein Degradation of the Ca2+-Activated K+ Channel KCa1.1 by Androgen Receptor Inhibition in Human Breast Cancer Cells
Source: Front Physiol. 2018 Apr 16;9:312. doi: 10.3389/fphys.2018.00312 (PMC5911984; doi:10.3389/fphys.2018.00312)

Supplementary Figure legends

Supplementary Figure S1. Relationship between expression levels of $K_{Ca}1.1$ and AR in human breast tumor tissues, PAX-induced depolarization responses in MDA-MB-453, YMB-1, and MDA-MB-231 cells, and effects of the siRNA-mediated blockade of AR on the expression levels of AR and $K_{Ca}1.1$ transcripts in MDA-MB-453 cells. A: The expression of AR and $K_{Ca}1.1$ transcripts in human breast tumor tissues was plotted (n=8). The dotted line shows the best fitting line, and the 'r' value shows the correlation coefficient for the linear fit between the variable X-axis and variable Y-axis. B: Summarized data are shown as the PAX-induced Δ relative fluorescence intensity of $DiBAC_4(3)$ in MDA-MB-453, YMB-1, and MDA-MB-231 cells. Cells were obtained from three different batches (48, 30, and 44 cells in each group). C, D: Real-time PCR assay for AR (C) and $K_{Ca}1.1$ (D) in control siRNA (si-cont)- and AR siRNA (si-AR)-transfected (for 72 hr) MDA-MB-453 cells (n=4 for each). Results were expressed as means ± SEM. *, **: $p<0.05$, 0.01 vs. si-cont.

Supplementary Figure S2. Effects of treatments with antiandrogens on expression levels of $K_{Ca}1.1$ regulatory β and γ subunit transcripts in MDA-MB-453 cells. Real-time PCR assays for KCNMB1 (A), KCNMB2 (B), KCNMB3 (C), KCNMB4 (D), LRRC38 (E), LRRC52 (F), and LRRC55 (G) in vehicle-, 1 μM BCT-, and 1 μM EZT-treated MDA-MB-453 cells (n=4 for each). Expression levels were expressed as a ratio to ACTB. Results are expressed as means ± SEM.

Supplementary Figure S3. Expression of AR, $K_{Ca}1.1$, LRRC26, LRRC38, LRRC52, and LRRC55 transcripts in the primary breast tumor (primary) and corresponding metastatic breast tumor (metastatic) of the same donor (a 65-year-old female). Real-time PCR assays for AR (A), $K_{Ca}1.1$ (B), LRRC26 (C), LRRC38 (D), LRRC52 (E), and LRRC55 (F) in 'primary' and 'metastatic' groups (n=3 for each). Expression levels were expressed as a ratio to ACTB. Results are expressed as means ± SEM.

Supplementary Figure S4. Effects of mTOR and AKT inhibitors on the viability and expression levels of AR transcripts in MDA-MB-453 cells. A, B: Effects of the treatment with everolimus (10 nM) (n=5) (A) and AZD5363 (1 μM) (n=3) (B) for 24 hr on the viability of MDA-MB-453 cells. Cell viability in the vehicle-treated group is arbitrary expressed as 1.0, and data are shown as 'relative cell viability'. C-D: Effects of the treatment with everolimus (10 nM) (C) and AZD5363 (1 μM) (D) for 24 hr on the expression levels of AR transcripts in MDA-MB-453 cells. Real-time PCR assays for AR in everolimus and AZD5363-treated MDA-MB-453 cells. Expression levels were expressed as a ratio to ACTB (n=4 for each). Results were expressed as means ± SEM. **: $p<0.01$ vs. the vehicle control.

Supplementary Figure S5. Effects of treatments with antiandrogens on expression levels of ubiquitin E3 ligases, NEDD4-1 and NEDD4-2 transcripts in MDA-MB-453 cells. A, B: Real-time PCR assays for NEDD4-1 (A) and NEDD4-2 (B) in vehicle-, 1 μM BCT-, and 1 μM EZT-treated MDA-MB-453 cells (n=4 for each). Expression levels were expressed as a ratio to ACTB. Results are expressed as means ± SEM.

Supplementary Figure S6. Effects of treatments with antiandrogens for 12 and 24 hr on expression levels of FBW7, MDM2, and MDM4 transcripts in MDA-MB-453 cells. Real-time PCR assays for FBW7 (A, B), MDM2 (C, D), and MDM4 (E, F) in antiandrogen-treated MDA-MB-453 cells for 12 (A, C, E) and 24 (B, D, F) hr. Expression levels were

expressed as a ratio to ACTB (n=4 for each). Results are expressed as means ± SEM. *, **: $p<0.05$, 0.01 vs. the vehicle control.

Supplementary Figure S7. Effects of the siRNA-mediated blockade of ubiquitin E3 ligases, FBW7, MDM2, and MDM4 on expression levels of FBW7, MDM2, and MDM4 transcripts in MDA-MB-453 cells, respectively. Real-time PCR assays for FBW7 (A), MDM2 (B), and MDM4 (C) in control siRNA (si-cont) and respective siRNA-transfected MDA-MB-453 cells for 72 hr (n=4 for each). Expression levels were expressed as a ratio to ACTB. Results are expressed as means ± SEM. **: $p<0.01$ vs. si-cont.

Supplementary Figure S8. Effects of the pharmacological blockade of ubiquitin E3 ligases on expression levels of $K_{Ca}1.1$ proteins and 1 μM PAX-induced depolarization responses in MDA-MB-453 cells. A: Effects of 10 μM nutlin-3a (MDM2/MDM4 inhibitor) and 20 μM SJ172550 (a selective MDM4 inhibitor) on the expression levels of $K_{Ca}1.1$ proteins in antiandrogen-treated MDA-MB-453 cells. Nutlin-3a and SJ172550 were applied 36 hr after the treatment with antiandrogens. Protein lysates of nutlin-3a (A)- and SJ172550 (B)-treated MDA-MB-453 cells were probed by immunoblotting with anti-$K_{Ca}1.1$ (upper panel) and anti-ACTB (lower panel) antibodies on the same filter. C, D: Summarized data are shown as the PAX-induced Δ relative fluorescence intensity of $DiBAC_4(3)$ in vehicle-, BCT-, and EZT-treated MDA-MB-453 cells. A total of 10 μM Nutlin-3a (C) or 20 μM SJ172550 (D) was applied 36 hr after the treatment with antiandrogens. Cells were obtained from (C) three-four different batches (94, 85, and 68 cells in each group) and (D) three different batches (50, 50, and 52 cells in each group). Results were expressed as means ± SEM. **: $p<0.01$ vs. the vehicle control.

Supplementary Figure S9. Effects of the STAT3 inhibitor, 5,15-DPP on the viability and expression levels of AR and $K_{Ca}1.1$ transcripts in MDA-MB-453 cells. A: Effects of the treatment with 5,15-DPP (10 μM) for 24 hr on the viability of MDA-MB-453 cells. Cell viability in the vehicle-treated group is arbitrary expressed as 1.0, and data are shown as 'relative cell viability' (n=3). B, C: Effects of the treatment with 5,15-DPP (10 μM) for 24 hr on the expression levels of AR (B) and $K_{Ca}1.1$ (C) transcripts in MDA-MB-453 cells. Real-time PCR assays for AR and $K_{Ca}1.1$ in 5,15-DPP-treated MDA-MB-453 cells. Expression levels were expressed as a ratio to ACTB (n=4 for each). Results were expressed as means ± SEM. **: $p<0.01$ vs. the vehicle control.

supplementary Fig. S1

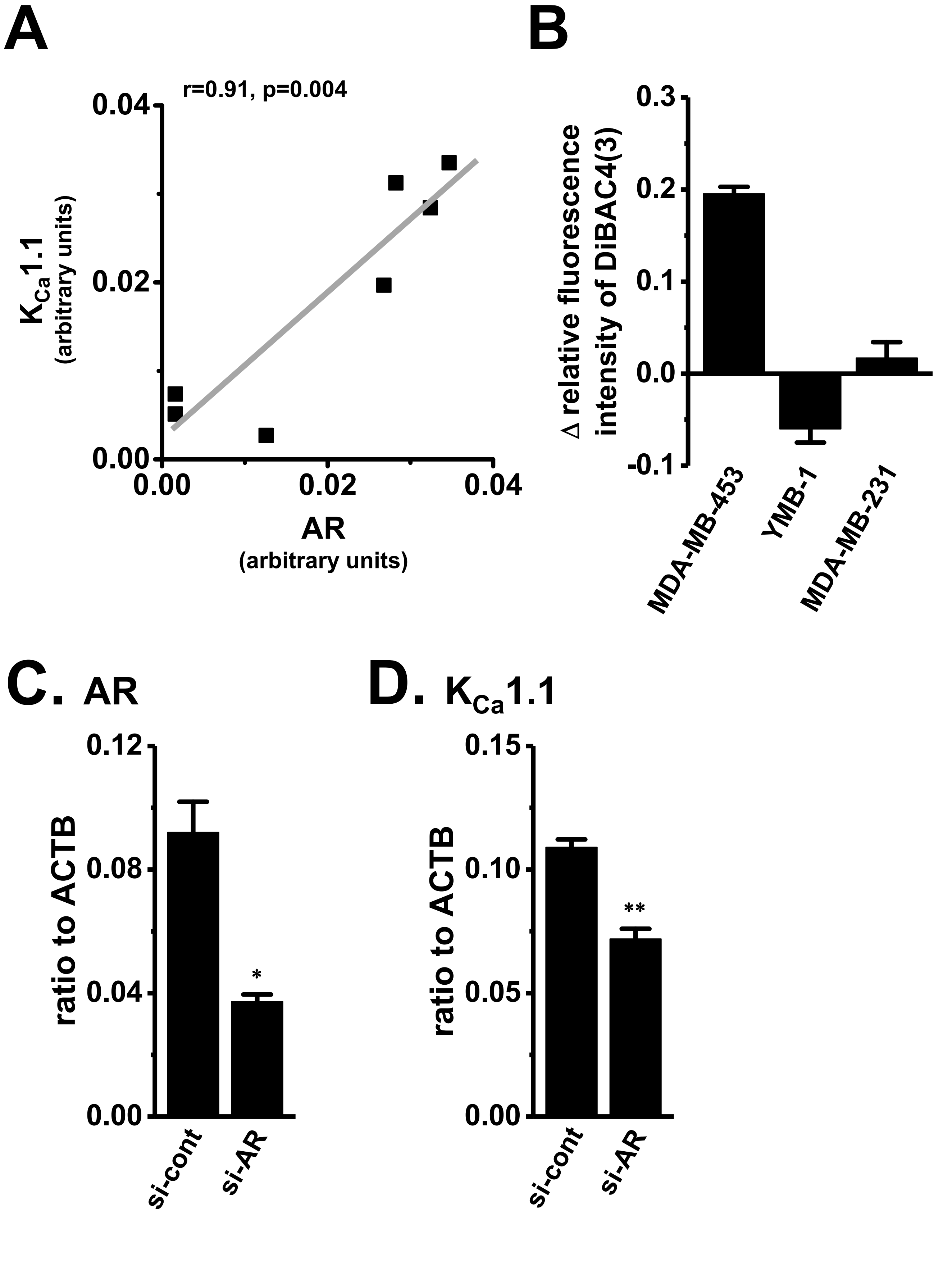

supplementary Fig. S2

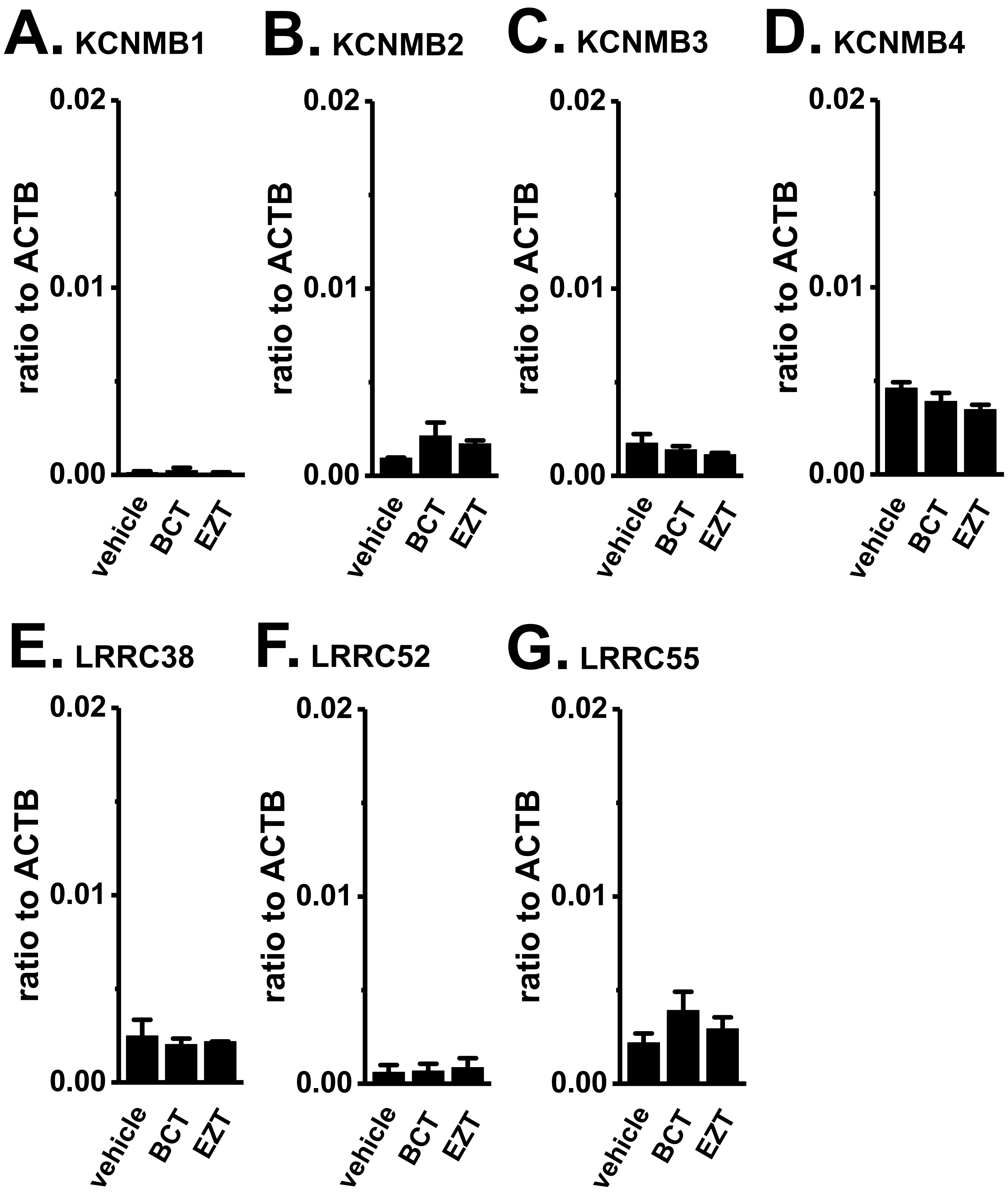

supplementary Fig. S3

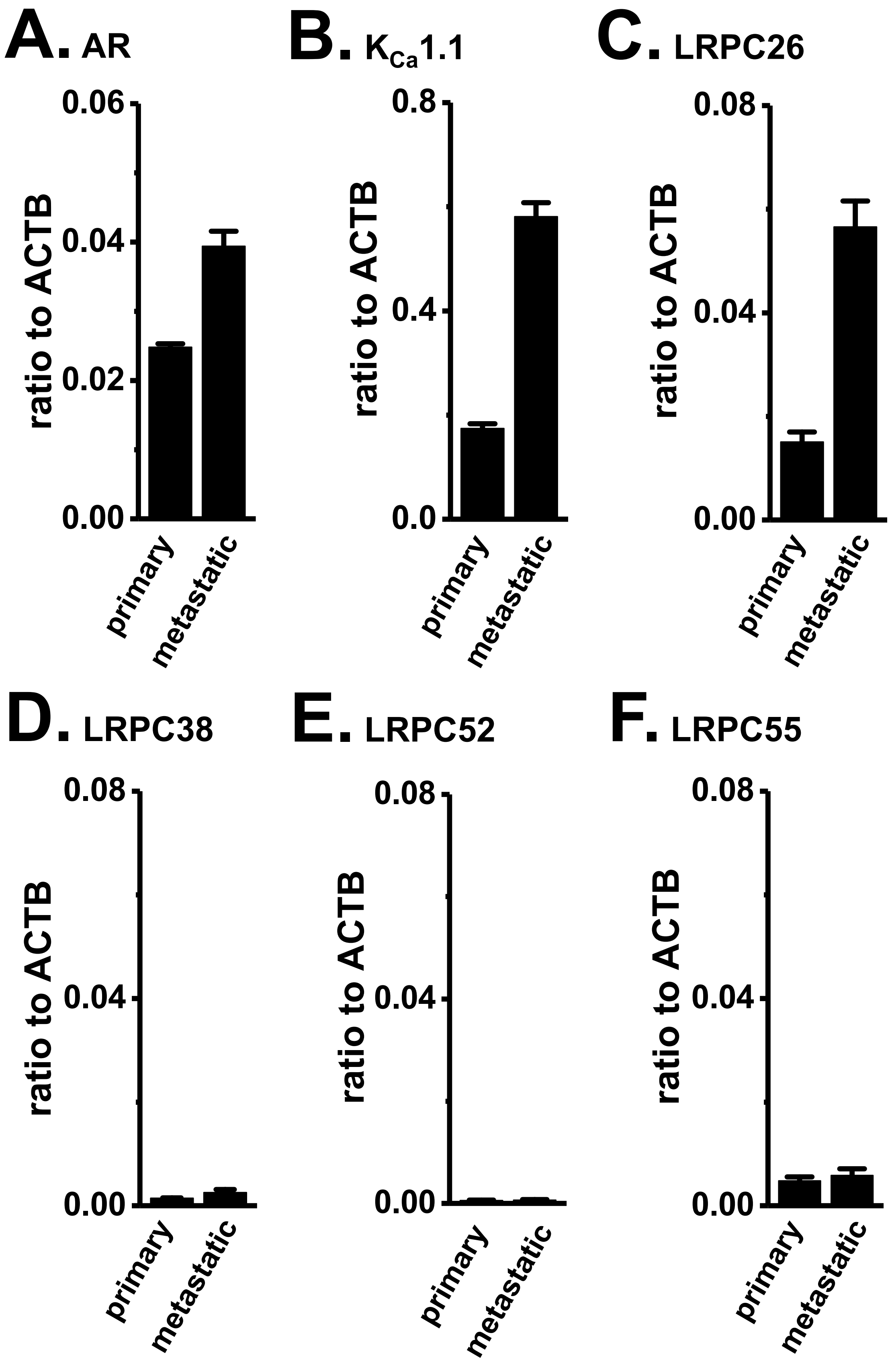

supplementary Fig. S4

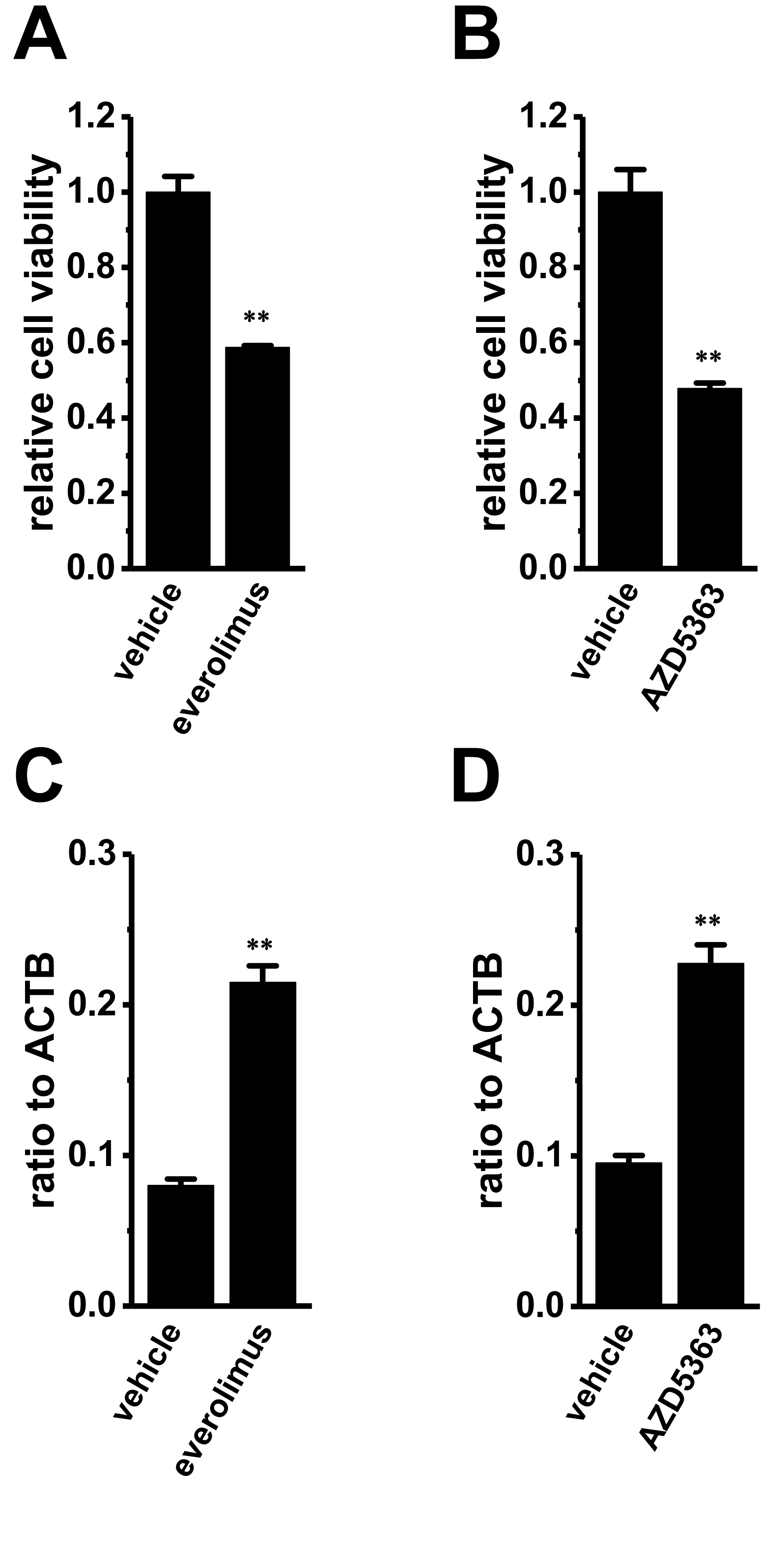

supplementary Fig. S5

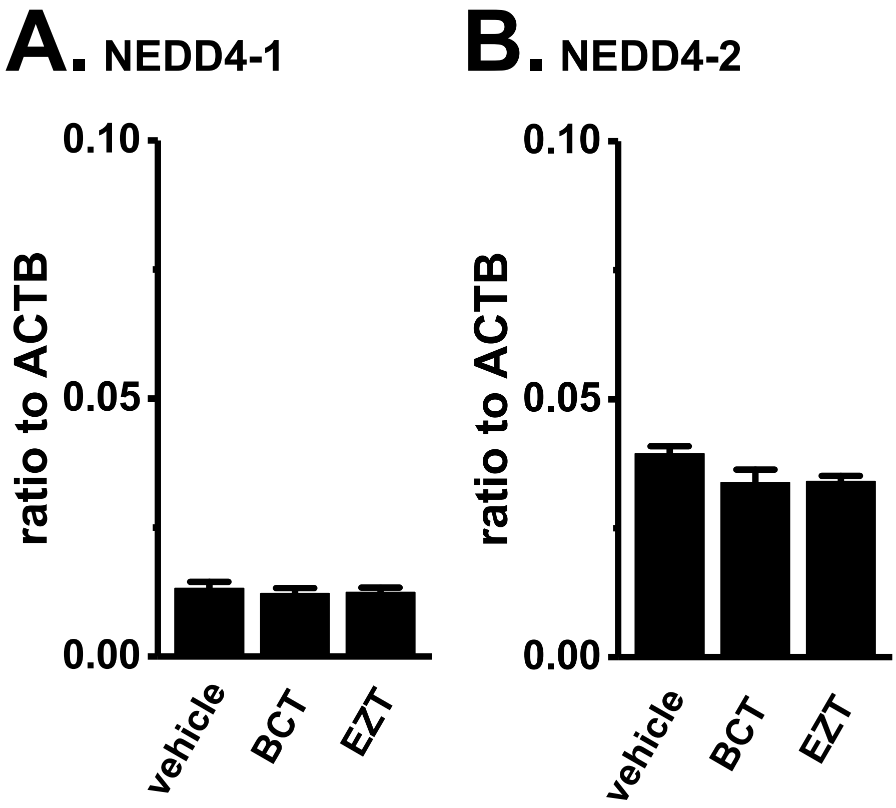

supplementary Fig. S6

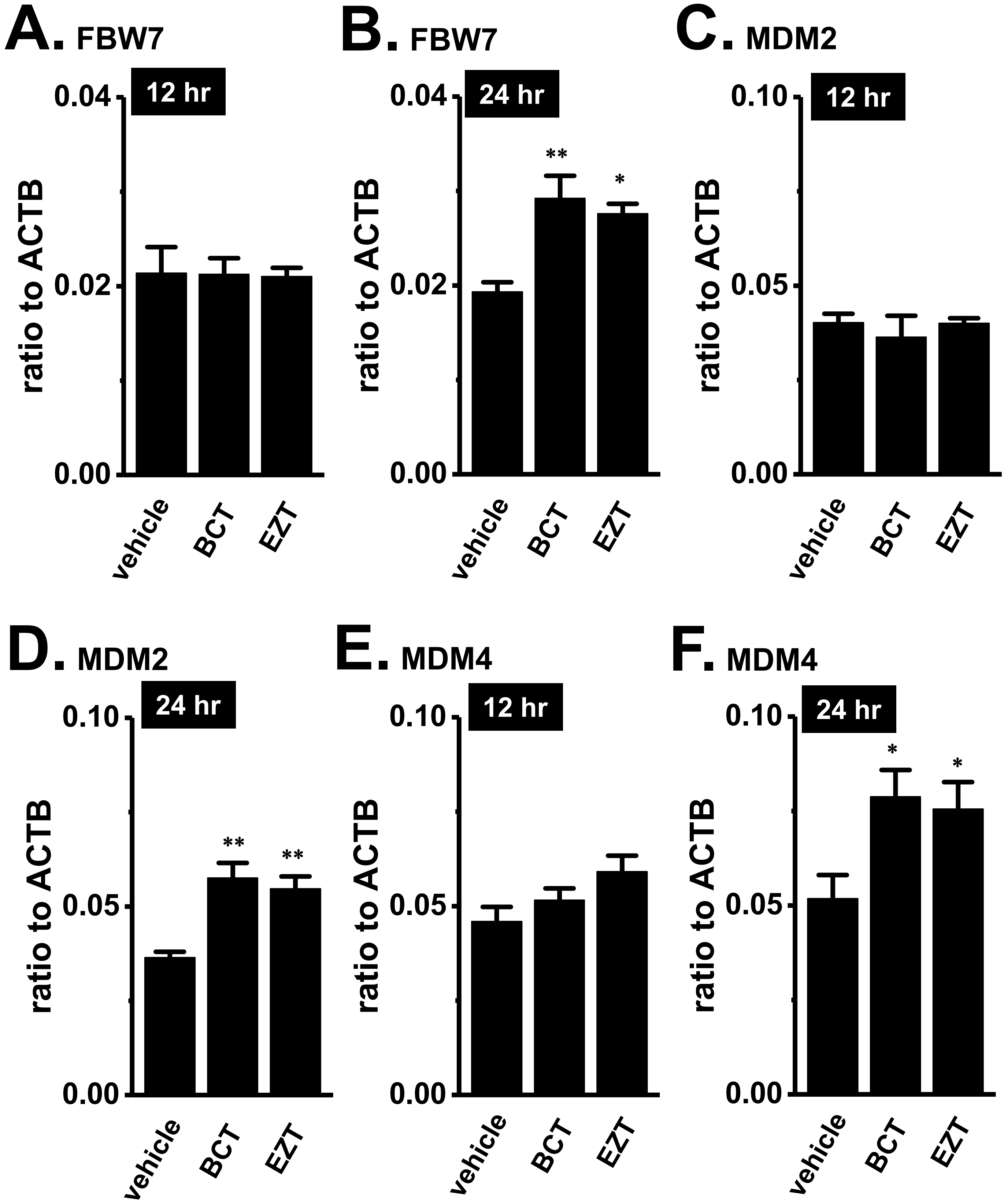

supplementary Fig. S7

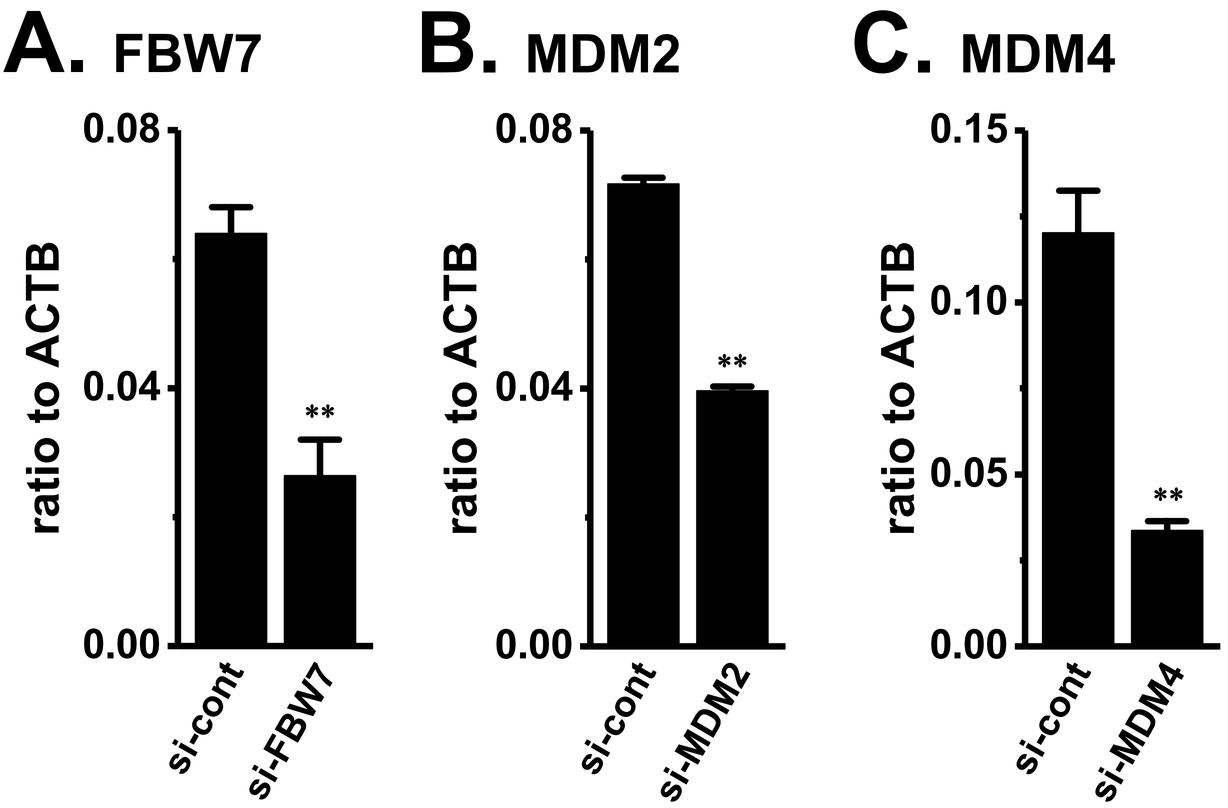

supplementary Fig. S8

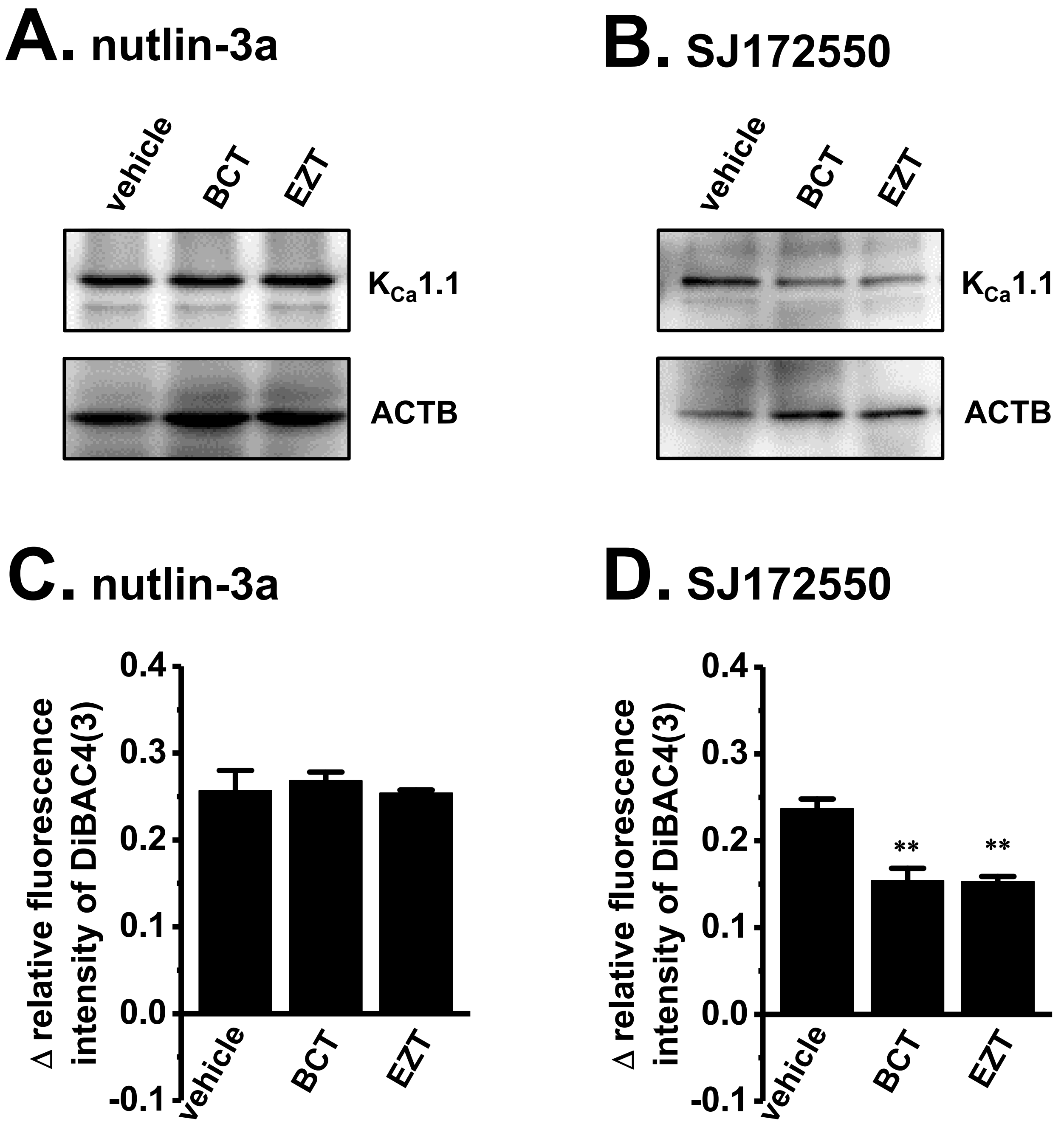

supplementary Fig. S9

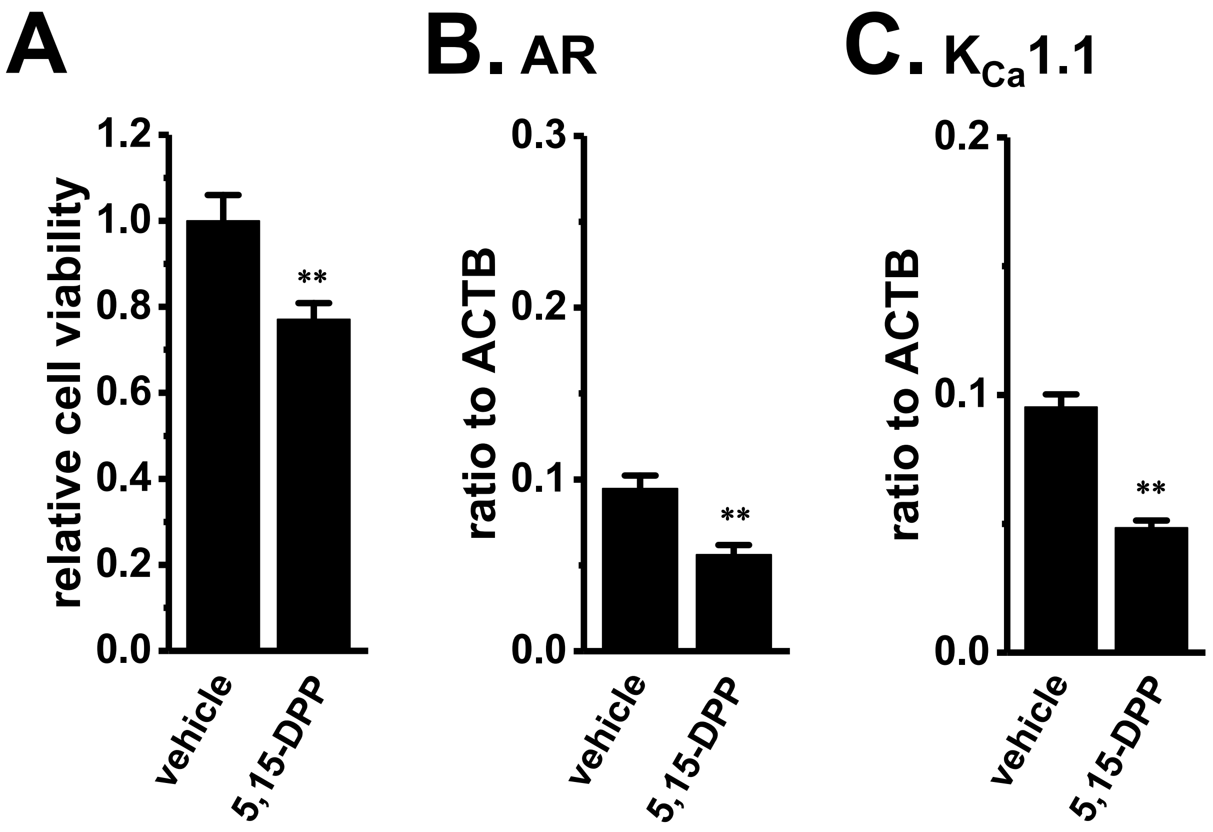

Supplement: Supplementary file 1 [file DataSheet1.PDF]
