# Supplementary material for: Transcriptional Repression and Protein Degradation of the Ca2+-Activated K+ Channel KCa1.1 by Androgen Receptor Inhibition in Human Breast Cancer Cells
Source: Front Physiol. 2018 Apr 16;9:312. doi: 10.3389/fphys.2018.00312 (PMC5911984; doi:10.3389/fphys.2018.00312)

Fig. 1B

1. MDA-MB-453
2. YMB-1
3. MCF-7
4. BT549

M. molecular marker

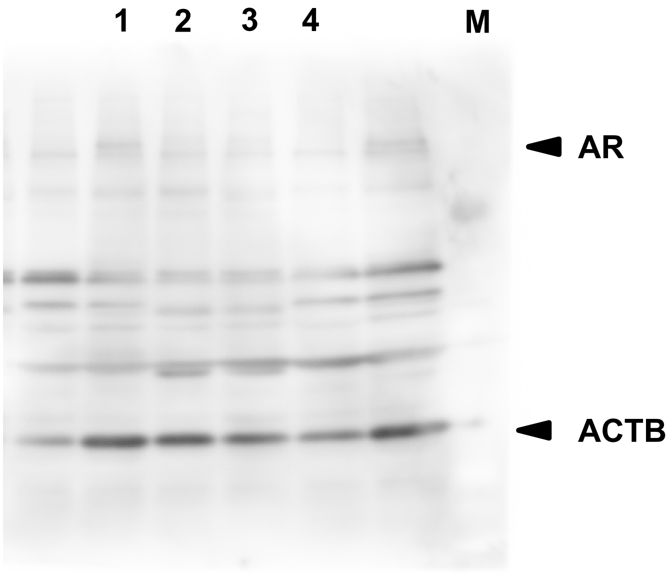

Fig. 4C

1. vehicle
2. BCT
3. EZT

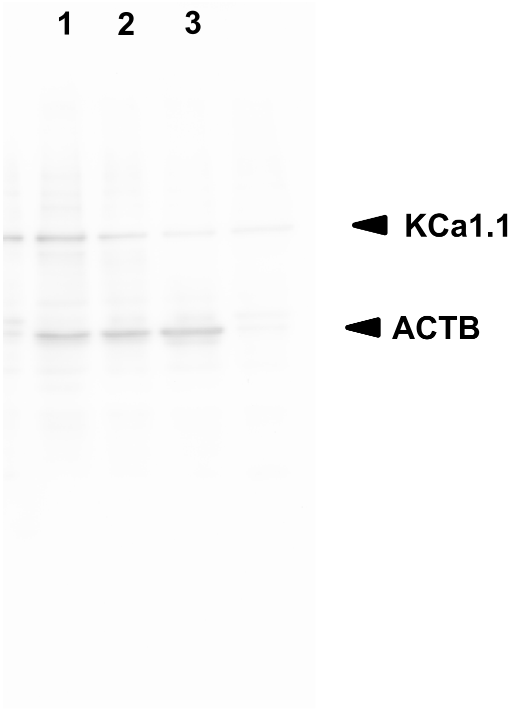

Fig. 5B

1. vehicle
2. BCT
3. EZT

M. molecular marker

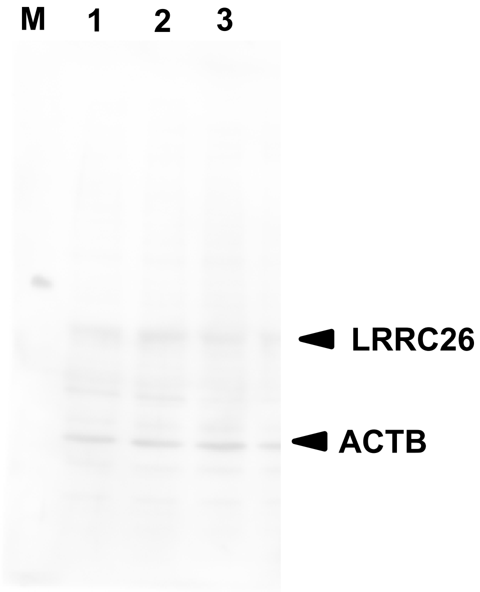

Fig. 6A

1. vehicle
2. BCT
3. EZT

M. molecular marker

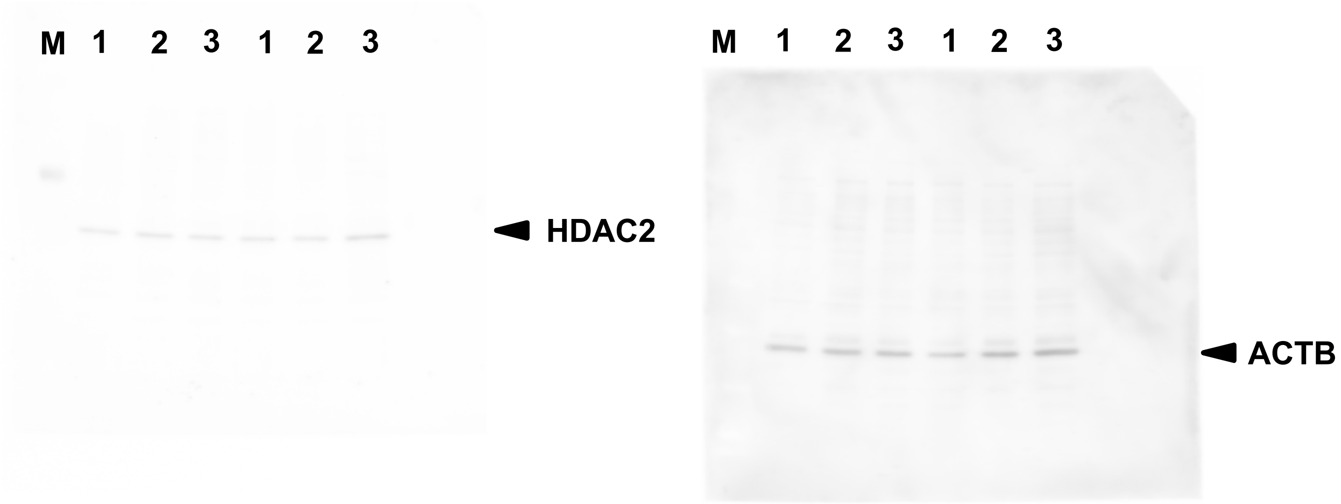

Fig. 7A

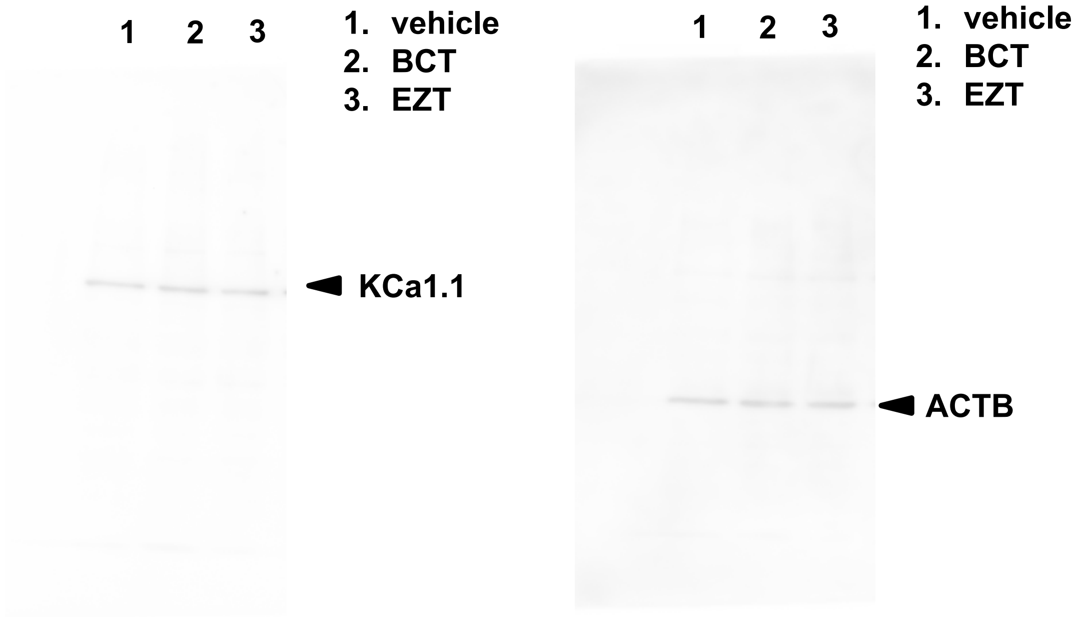
1 2 3
1. vehicle
2. BCT
3. EZT
KCa1.1
1 2 3
1. vehicle
2. BCT
3. EZT
ACTB

Fig. 8D

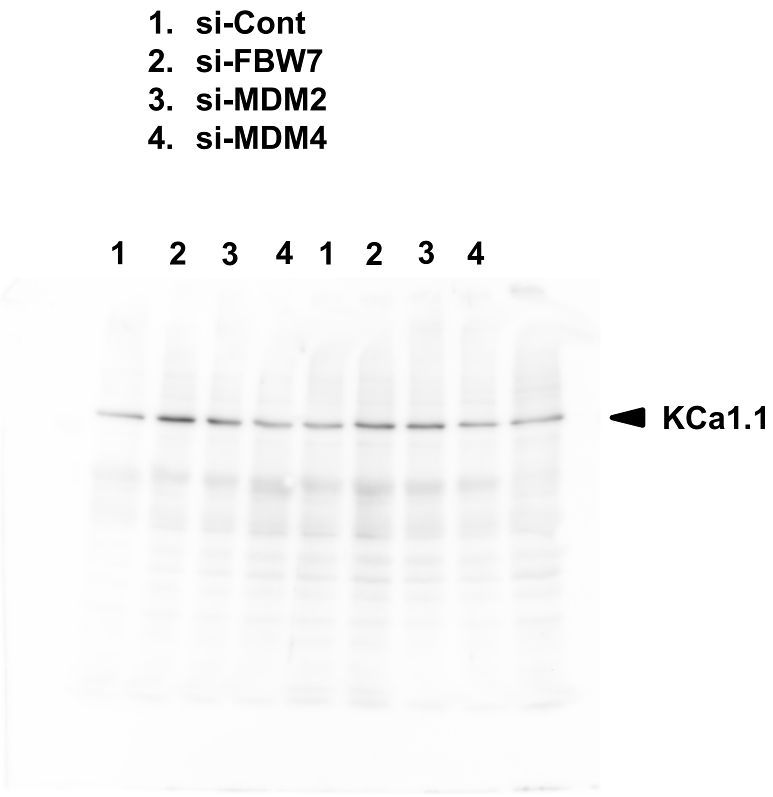
1. si-Cont
2. si-FBW7
3. si-MDM2
4. si-MDM4
1 2 3 4 1 2 3 4
KCa1.1

Supplementary Fig. S8A

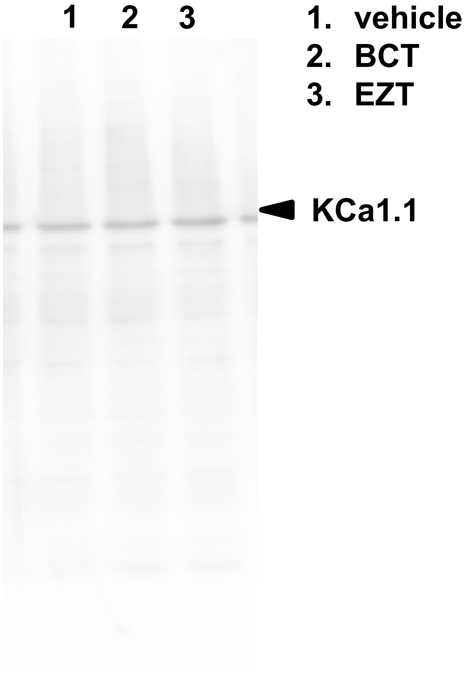

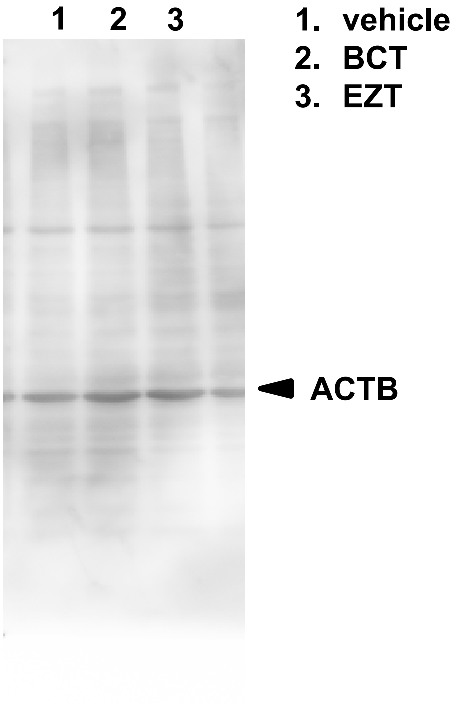

Supplementary Fig. S8B

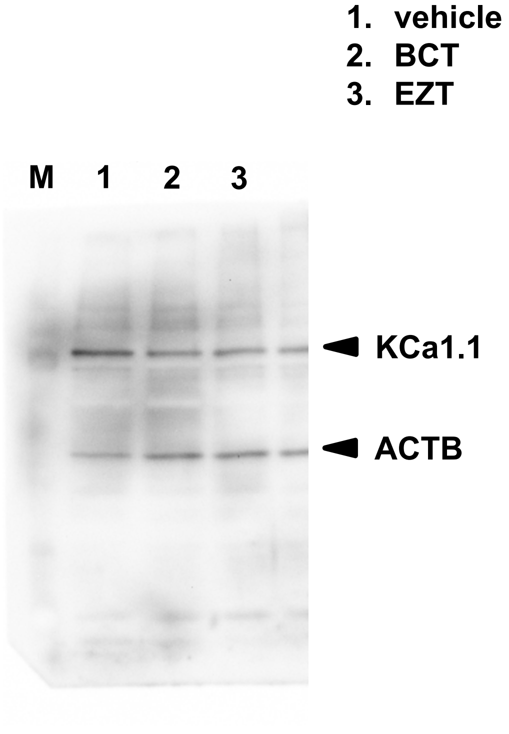

Supplement: Supplementary file 2 [file DataSheet2.PDF]
